# Exploring the Structural Dynamics of LeuT Using EPR Spectroscopy: A Focus on Transmembrane Helix 10

**DOI:** 10.1111/jnc.70034

**Published:** 2025-03-07

**Authors:** Petros Tsalagradas, Callum Eke, Courtney Andrews, Fraser MacMillan

**Affiliations:** ^1^ Henry Wellcome Unit for Biological EPR, School of Chemistry University of East Anglia Norwich UK

**Keywords:** dynamics, EPR, membrane transport protein, NSS, SLC6, spin label

## Abstract

The amino‐acid transporter LeuT from *Aquifex aeolicus* is a well‐studied bacterial homologue of the neurotransmitter: sodium symporters (NSS), especially the solute carrier 6 (SLC6) family. Within the nervous system, SLC6 transporters play a vital role in the termination of synaptic transmission, and their dysfunction leads to severe neurological conditions, rendering them key pharmacological targets. LeuT was the first SLC6 homologue to be crystallised and remains the main reference transporter to develop transport cycle models for its eukaryotic counterparts. Here, we aim to probe LeuT and investigate mechanistically important conformational changes using a combination of Site‐Directed Spin Labelling (SDSL) and Electron Paramagnetic Resonance (EPR) spectroscopic techniques in detergent solubilised micelles and proteoliposomes. We focus, primarily, on ‘subtle’ structural, molecular motions occurring at the extracellular region of transmembrane helix (TM) 10, which cannot be resolved using conventional high‐resolution crystallographic techniques. We observe similar but not identical ion/ligand‐dependent conformational changes of LeuT on the extracellular domain of TM10 in detergent micelles and proteoliposomes. Close agreement is also observed between *in silico* analysis of existing static structural models and the experimental data acquired here in the form of coarse‐grained accessibility restraints, demonstrating that such subtle movements can be important for understanding both function and mechanism. The observed differences for the dynamics of LeuT in different environments underpin future work, which aims to explore ‘more native’ reconstituted proteoliposome conditions more thoroughly using pulsed EPR methods before generalised conclusions can be drawn on the physiological relevance of such structural changes and whether they can provide novel insights on the molecular events underlying the transport cycle of LeuT.

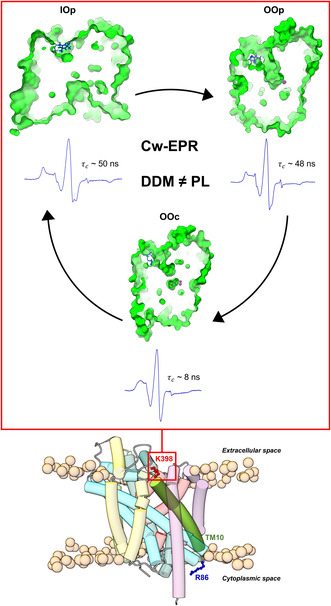

## Graphical abstract

Electron Paramagnetic Resonance (EPR) Spectroscopy is used to identify ‘subtle’ movement of transmembrane helix10 (TM10) of the bacterial amino–acid transporter, LeuT, not easily observed in static crystal structure models. The key findings on a single cysteine (K398C) variant, located in the extracellular region of TM10 at the membrane–solvent interface, are summarised. The molecular motion and accessibility of an exogenous nitroxide spin probe attached to K398C are probed using a combination of EPR spectroscopic methods. Ion/substrate–dependent structural rearrangements of TM10 are observed during the transport cycle, which are similar but not identical between detergent solubilised and proteoliposome reconstituted environments.

**Abbreviations**
ADHD: Attention deficit hyperactivity disorder
DEER: Double electron–electron resonance spectroscopy
EL: Extracellular Loop
EPR: Electron Paramagnetic Resonance spectroscopy
EV: Extracellular Vestibule
FRET: Förster resonance energy transfer
GABA: γ‐aminobutyric acid
GWAS: Genome‐wide association studies
HDX‐MS: Hydrogen/Deuterium eXchange Mass Spectrometry
IL: Intracellular Loop
IOc: Inward facing Occluded
IOcF: Inward facing Occluded with phenylalanine (PDB: 6XWM)
IOp: Inward facing Open
IOpApo: Inward facing Open Apo (PDB: 3TT3)
LeuT: Leucine Transporter
MD: Molecular Dynamics
NSS: Neurotransmitter: sodium symporters
OCD: Obsessive compulsive disorder
OOc: Outward facing Occluded
OOcA: Outward facing Occluded with alanine (PDB: 3F48)
OOcL: Outward facing Occluded with leucine (PDB: 2A65)
OOp: Outward facing Open (PDB: 3TT1)
OOpW: Outward facing Open with tryptophan (PDB: 3F3A)
OPM: Orientations of Proteins in Membranes
OReApo: Outward facing Return Intermediate Apo (PDB: 5JAE)
PDB: Protein Data Bank
RRID: Research Resource Identifier (see scicrunch.org)
SDSL: Site‐directed spin labeling
SLC6: Solute Carrier 6
SPA: Scintillation Proximity Assay
SSRIs: Selective serotonin reuptake inhibitors
TCAs: Tricyclic antidepressants
TM: Transmembrane Helix
TS: Tourette's Syndrome

## 1 Introduction

Neurotransmitter sodium symporters (NSSs) comprise a group of secondary active transporters that belong to the solute carrier 6 (SLC6) family of membrane transporters. SLC6 members include both eukaryotic and prokaryotic transmembrane proteins whose principal function requires utilization of an electrochemical gradient of Na^+^ ions across plasma membranes, thus catalyzing the otherwise thermodynamically unfavorable reuptake of a variety of molecules, including amino acids and γ‐aminobutyric acid (GABA), as well as biogenic amines such as norepinephrine, serotonin, and dopamine into the presynaptic neuron at the synaptic cleft and thereby regulating neurotransmission and homeostasis in neuronal synapses (Rudnick et al. 2014; Kristensen et al. 2011). Previous genome‐wide association studies (GWAS) on the *SLC6* genes highlighted statistically strong associations between the dysfunction of several NSSs and the development of behavioral, neuropsychiatric, and neurodegenerative disorders, such as with the *SLC6A4* gene being linked to the development of autism, obsessive‐compulsive disorder (OCD), Tourette's syndrome (TS), and clinical depression (Coutinho et al. 2004; Voyiaziakis et al. 2011; Moya et al. 2013; Miozzo et al. 2020). Overall, NSS function impairment has been linked with the development of schizophrenia, Parkinson's disease, attention deficit hyperactivity disorder (ADHD), epilepsy, and even metabolic disorders (Rudnick et al. 2014; Hahn and Blakely 2007; Adams and Defelice 2002; César‐Razquin et al. 2015; Pramod et al. 2013; Broer and Gether 2012; Le et al. 2024; Wang et al. 2020; Cordeiro et al. 2010). These important associations of NSS dysregulation with disease phenotypes, as well as the fact that NSSs are receptors for psychostimulants such as the illegal drug cocaine, have made this class of proteins a desirable biopharmaceutical drug target (César‐Razquin et al. 2015) while they currently function as targets for several antidepressant therapeutic strategies such as selective serotonin reuptake inhibitors (SSRIs) (Edinoff et al. 2021) and tricyclic antidepressants (TCAs) (Moraczewski et al. 2024).

**FIGURE 1:**
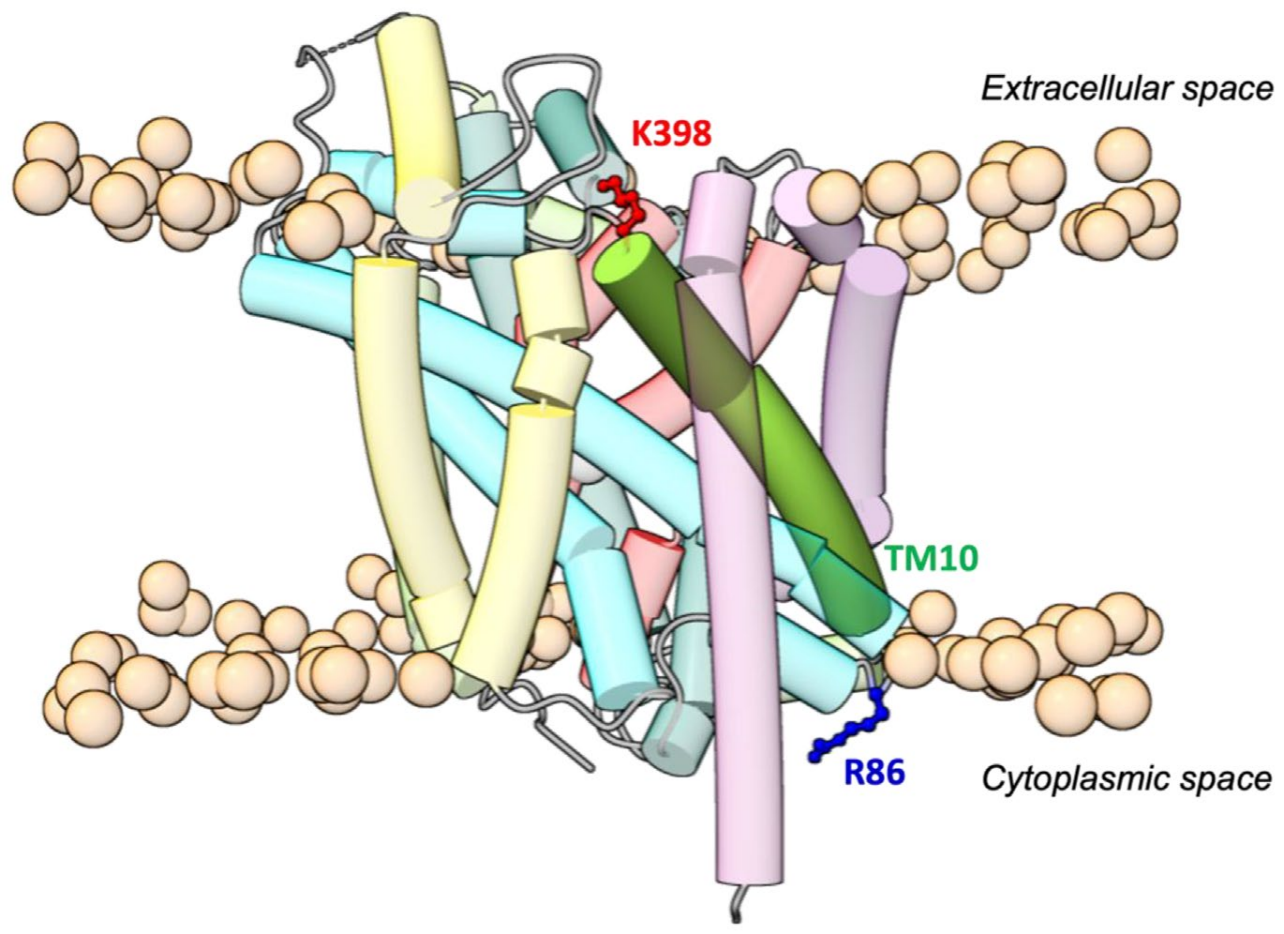
Visualisation of residues K398 and R86 on LeuT. The residues K398 (Lys398) and R86 (Arg86) are highlighted (ball and stick) in red and blue, respectively, to indicate their localisation relative to the membrane plane (illustrated by phospholipids' heads depicted as orange atoms) and environment (extracellular or cytoplasmic). TM10 is highlighted, while all other structural elements of the LeuT model in the outward–facing occluded conformation (*OOcL*, PDB: 2a65) have been set to transparent for clarity. The LeuT crystal structure model was visualised in ‘UCSF ChimeraX’ (Pettersen et al. 2021). *OOcL*, Outward facing Occluded with leucine (Na^+^/leucine‐bound).

The use of bacterial homologues of membrane proteins for the interpretation of structural and functional data has proven a fruitful and hence common strategy for elucidating both structural and mechanistic information for eukaryotic NSSs. In particular, the bacterial Leucine Transporter (LeuT), a small amino acid symporter originating from *Aquifex aeolicus*, which couples the intracellular transport of Na^+^ ions with substrate transport, has emerged as one of the most utilised and powerful models for structural and more recent biophysical mechanistic studies of its eukaryotic counterparts since its three‐dimensional structure was first determined by x‐ray crystallography 20 years ago (Yamashita et al. 2005). In this previous study, Yamashita et al. identified a novel topology, revealing that LeuT is composed of 12 transmembrane helices (TM), with TM1‐5 being related to TM6‐10 via a pseudo‐2‐fold symmetry (Yamashita et al. 2005) (Figure 1). This unique protein fold was eventually shown to be conserved across several other Na^+^‐dependent membrane transporters (Kazmier et al. 2017), despite their relatively low (~20%) sequence identity (Thijs et al. 2006), further validating LeuT's use as a suitable model for studying the transport mechanisms of eukaryotic NSS homologues.

Currently, the most accepted LeuT transport mechanism (‘*rocking bundle*’) is derived primarily from static structural information obtained from X‐ray crystallographic data which capture LeuT in different static ‘conformational snapshots’ during its transport cycle (Kazmier et al. 2017; Forrest and Rudnick 2009). Under physiological conditions, LeuT is proposed to predominantly adopt an ‘*outward facing open'* (*OOp*) conformation (PDB entry 3TT1), stabilised by the binding of two sodium (Na^+^) ions (Krishnamurthy and Gouaux 2012) (Figure S1A). In this conformation, the primary substrate binding site (S1) is located in a cavity formed by the partial unwound TM helices 1 and 6 within the central pore of the transporter (Navratna and Gouaux 2019). Subsequent substrate binding induces conformational changes in the bundle domain (TM1, 2, 6, 7), resulting in movement to partially close both the protein's extracellular vestibule (EV) and S1, and thereby adopting the ‘*Outward facing Occluded with leucine*’ (*OOcL*) (PDB entry 2A65) conformation (Yamashita et al. 2005) (Figures S1B). The protein then transitions towards an ‘*Inward facing Occluded with phenylalanine*’ (*IOcF*) (PDB entry 6XWM) conformation (Gotfryd et al. 2020) (Figure S1C), with this step possibly being aided by the further binding of substrate molecules to a secondary binding site (S2), although this step has been constantly and critically debated within the research community for over a decade (Shi et al. 2008; Quick et al. 2009; Quick et al. 2012; Gedeon et al. 2010; Zhao et al. 2011; Wang et al. 2012; Khelashvili et al. 2013; Reyes and Tavoulari 2011; Tavoulari et al. 2016; Zhang et al. 2018). TMs 1b, 2, 6a and 7 undergo further conformational rearrangements, bunching together, thus completely sealing the EV (Malinauskaite et al. 2014) and partially unwinding TM5 in the process, which results in the Na^+^ binding sites becoming exposed to the cytoplasm (Merkle et al. 2018). Such structural alterations induce the intracellular release of Na^+^ ions that significantly shift TM1a and cause subtle movements of TM2, 7 and 6b (Krishnamurthy and Gouaux 2012). Finally, LeuT adopts the ‘*Inward facing Open Apo*’ conformation (*IOpApo*) (PDB entry 3TT3) with S1 and the bound substrates now exposed to the cell's interior (Krishnamurthy and Gouaux 2012) (Figure S1D). Substrate is then released ‘resetting’ LeuT to its *OOp* conformation by transitioning through a transient and yet to be structurally resolved, ‘*Outward facing Return Intermediate Apo*’ (*OReApo*) (PDB entry 5JAE) (Figure S1E) state, before two sodium ions rebind stabilising the *OOp* state, thereby completing the transport cycle (Malinauskaite et al. 2016).

Despite the wealth of static crystallographic information, the current understanding of the dynamics that govern these conformational changes during the transport cycle remains limited and is mostly inferred from molecular dynamics (MD) simulations (Forrest and Rudnick 2009; Khelashvili et al. 2013; Khelashvili et al. 2016; Chen and Chung 2015; Gur et al. 2015; Thomas et al. 2012; Shi et al. 2008; Shi and Weinstein 2010; Li et al. 2019; Noskov and Roux 2008; Jørgensen et al. 2007). Current trends in structural biology suggest that static crystal structures of dynamic membrane proteins such as LeuT provide the majority of the structural and thus mechanistic insights into dynamic transport mechanisms, which can be called into question in terms of their physiological relevance and lack of dynamic information (Zheng et al. 2015), especially for structures containing structural mutations, highlighting the significance of more experimental data that focus on these structural dynamics. This increases the demand to further verify the structural details and data pertaining to dynamic movements associated with such conformational steps under more native experimental conditions or by alternative approaches such as electron paramagnetic resonance (EPR) spectroscopy (Prisner et al. 2001; Schiemann and Prisner 2007; Sahu and Lorigan 2015; Sahu and Lorigan 2020; Sahu et al. 2013).

EPR spectroscopy has long been a tool utilized in the investigation of the structure and mechanism of membrane proteins containing intrinsic paramagnetic centers. Notably, EPR has provided significant insight into the investigation of the mechanism for electron transfer in both bacterial and mitochondrial electron transport chains for over 60 years (Cammack and MacMillan 2010; MacMillan 2005). Despite the sensitivity and precision provided by EPR, its application to biological systems has often been thought to be restricted due to the limited number of intrinsic paramagnetic target molecules. It was not until the establishment of a site‐directed spin labeling (SDSL) methodology, the attachment of paramagnetic labels to specific positions on a protein, pioneered by McConnell and Hubbell (Hubbell and McConnell 1971) that EPR quickly expanded to cover a much wider range of biomolecules. The recent development of SDSL‐EPR, particularly methodology employing nitroxide‐based spin labels, has proved a significant boon for the study of membrane proteins allowing for example: the extraction of dynamic information from EPR line shape analysis (Klug and Feix 2008); topological information with respect to membranes via interpretation of EPR power saturation data (Hubbell and Altenbach 1994); and, especially, coarse‐grained structural information in the form of distance restraints from double‐electron electron resonance (DEER) EPR techniques (Jeschke 2012). Previous work by Claxton et al. (2010) and Kazmier et al. (2014a) has indicated the opportunities for combining SDSL and EPR on LeuT to access both global and local conformational changes, suggesting novel dynamic structural insights on the LeuT transport mechanism are possible.

Here SDSL, in combination with both experimental and *in silico* EPR methods, is used to investigate the structural dynamics of LeuT's EV in distinct conformational configurations both in detergent‐solubilised micelles and especially in the more physiologically relevant, native membrane mimicking environment formed by LeuT reconstitution into proteoliposomes (Claxton et al. 2010; Kazmier et al. 2014a). Specifically, the extracellular region of TM10, located on the lipid membrane‐aqueous solvent interface (K398) (Figure 1) was investigated regarding its structural flexibility during the transport cycle's induced conformational changes. Initially, *in silico* EPR analysis was employed to visualise and computationally attach an exogenous EPR spin label (MTSL) to a LeuT variant (K398C) determining this position's suitability for further investigation with experimental EPR approaches. Subsequently, LeuT K398C was successfully purified and efficiently spin labelled before either direct investigation with EPR in detergent micelles or after reconstitution into proteoliposomes. The study investigated the spin label's accessibility at ambient temperature under several varied ion/substrate‐dependent conformational states of the transport cycle. Finally, data analysis delineated and quantified various EPR spectral components that describe intermediate protein conformations not easily detected using other biophysical techniques to describe the dynamics of K398 implicated in the role of TM10 during transport. Identical EPR spectroscopic measurements on the LeuT variant R86C, located on a rigid, connecting region between IL1 and the start of TM3 (scaffold domain) in the cytoplasmic space (Figure 1) served as a static control.

## 2 Materials and Methods

### 2.1 Plasmids

The genes encoding the LeuT single–cysteine variants, K398C (supplied by Prof Claus Løland, University of Copenhagen, Denmark) and R86C (*de novo* designed at Medical University of Vienna, Austria) (Khan et al. 2020), were incorporated into a pET16b derivative plasmid harboring a carboxy (C)‐terminal octahistidine tag and a thrombin site via site‐directed mutagenesis (Billesbolle et al. 2016). Their sequences were confirmed by DNA sequencing (data not shown).

### 2.2 Expression, Purification, and Spin Labelling of LeuT K398C

See the Supporting Information—Data S1 for the experimental details on the LeuT expression, purification, and spin labeling methodology. Both LeuT cysteine mutants, K398C and R86C, were overexpressed, purified, and spin–labelled using identical experimental procedures.

### 2.3 Reconstitution of Spin Labelled LeuT K398C Into Proteoliposomes

Purified spin‐labelled LeuT K398C was concentrated to approximately 120 μM in IMAC buffer. Initially, *Escherichia coli* total lipid extract (17.5 mg, Avanti Polar Lipids Inc., RRID:SCR_016391, cat. no. 100500) was dried under a constant steady argon stream for at least 30 min to form a thin lipid film and to remove the solvent (CHCl_3_). Dried lipids were rehydrated in buffer A (20 mM HEPES pH: 7.5, 100 mM KCl) to a final lipid concentration of 20 mg mL^−1^ followed by immediate gas exchange (O_2_ ➔ Argon) for 2 min. The lipid resuspension was subjected to 7 freeze–thaw cycles using liquid nitrogen, thawing at room temperature, leading to the formation of large multilamellar vesicles (LMVs). Subsequently, the lipid mixture was extruded 15 times through pre‐equilibrated polycarbonate filter membranes with buffer A and a pore size of 400 nm (Nucleopore Polycarbonate Track‐Etch Membrane, Whatman, Cytiva, cat. no. WHA10417106). The lipid extrusion resulted in the formation of large unilamellar vesicles (LUVs) which were then diluted to a final lipid concentration of 4 mg mL^−1^ using buffer B (20 mM HEPES pH: 7.5, 100 mM KCl, 0.05% DDM, 25% glycerol). Liposomes were destabilised by the addition of Triton X‐100 (150 μL per 5 mL of lipids, 10% w/v, Sigma Aldrich, cat. no. 93443) followed by the addition of spin‐labelled LeuT K398C at a 1:25 (w/w) reconstitution ratio. The mixture was gently mixed and incubated for 30 min at 4°C under gentle agitation. The detergent was removed by stepwise addition of pre‐equilibrated BioBeads SM‐2 (Bio‐Rad, cat. no. 1528920) in buffer A, repeated four times. Specifically, the proteoliposomes were mixed with 200 mg pre‐equilibrated BioBeads SM‐2 per 5 mL of sample and left to incubate at ambient temperature (298 K) for 30 min under gentle agitation. The same amount of BioBeads was added twice, and the mixture was incubated for 1 h at 4°C under gentle agitation at first, and then overnight under the same conditions. The next day, the process was repeated once more followed by a 1 h incubation at 4°C. BioBeads were removed by gravity filtration, and the proteoliposomes were diluted 10×‐fold in buffer A and collected through ultracentrifugation (~96 040 rcf for 2:30 h at 4°C). The pellet containing the reconstituted liposomes was carefully resuspended in buffer C (230 μL buffer A in deuterated water or D_2_O, Sigma Aldrich, cat. no. 151882) resulting in a final protein concentration of ~53 μM. The reconstituted liposomes with spin‐labelled LeuT K398C were aliquoted, flash‐frozen in liquid nitrogen, and stored at −80°C. This protocol, adapted from Hall et al. (2020), was also used to produce spin‐labelled R86C LeuT.

### 2.4 Continuous Wave Electron Paramagnetic Resonance Spectroscopy

Ambient temperature X‐band continuous‐wave (cw) EPR spectra of spin‐labelled LeuT were acquired using a *Bruker* eleXsys E560 spectrometer fitted with a *Bruker* ER4123D loop‐gap resonator. The total active sample volume was 8–10 μL in custom‐made glass capillaries (Drummond Scientific, cat. no. 9‐000‐1000). Experimental parameters for the acquisition of ambient temperature cw EPR spectra in both detergent‐solubilised micelles and reconstituted proteoliposomes were: microwave power, 0.2 mW; field modulation amplitude, 0.1–0.2 mT; field modulation frequency, 100 kHz; the number of accumulated scans varied from 40 to 70 depending on the signal‐to‐noise ratio. Labelling efficiency was quantified against a series of calibration standards (2,2,6,6‐tetramethylpiperidin‐1‐yl)oxidanyl (TEMPO, Sigma Aldrich, cat. no. 214000).

EPR experiments in both detergent micelles and proteoliposomes were performed on: the *IOpApo* conformation in the absence of Na^+^ ions and substrate; the *OOp Na* ^+^‐*bound* conformation formed by the addition of NaCl (200 mM, Sigma Aldrich, cat. no. S7653) followed by incubation (273 K, 1 h); the *OOcX* (where *X* = *A* for L‐alanine (Sigma Aldrich, cat. no. A7627) or *L* for L‐leucine (Sigma Aldrich, cat. no. L8000)) *Na* ^+^/*substrate‐bound* conformation and *OOpW* (W for L‐tryptophan, ThermoFisher scientific chemicals, cat. no. 10543881) formed by the successive addition of substrate (4× molar excess of substrate to protein) and NaCl (200 mM) followed by incubation (273 K, 1 h). This protocol was adapted from (Claxton et al. 2010; Kazmier et al. 2014a).

For low‐temperature EPR studies (Figure S4) K398C spin‐labeled LeuT (70 μM) was added to 4 mm suprasil quartz tubes (Wilmad SQ‐707; Wilmad‐LabGlass, Vinland, NJ, USA, Sigma Aldrich, cat. no. Z566535), poised in the respective state (*IOpApo*, *OOp*, *OOcL*, *OOcA*, *OOpW*) and flash frozen in liquid nitrogen. X‐band cw‐EPR spectra were recorded on a Bruker eleXsys E560 spectrometer using a cylindrical Bruker cavity (ER4122SHQE) equipped with an Oxford helium cryostat (ESR900). Experimental parameters: microwave power, 10 μW; field modulation amplitude, 0.1 mT; field modulation frequency, 100 kHz; temperatures, 10 and 50 K. Using identical experimental conditions and acquisition parameters, X‐band cw EPR spectra of spin‐labeled LeuT R86C in detergent–solubilized micelles were measured under the same conformational states at cryogenic temperatures (Figure S5). The measured spectra were corrected for an offset against a known g standard [DPPH, 1,1‐diphenyl‐2‐picrylhydrazyl, Bruker, *g* = 2.00360 ± 0.00002]. Spectral simulations were performed using the Matlab‐based Easyspin package.

### 2.5 Cw EPR Spin Label Accessibility Measurements

EPR microwave power saturation measurements were performed as above but using gas‐permeable TPX capillaries (Molecular Specialties Inc., cat. no. TPX‐2) in an inert N_2_ environment (active sample volume 3–4 μL) as described previously (Klug and Feix 2008; Altenbach et al. 1994; Altenbach et al. 1989; Altenbach et al. 1990). All conformational states (*IOpApo*, *OOp*, *OOcL, OOcA*, and *OOpW*) in both detergent‐solubilised micelles and reconstituted proteoliposomes were studied after equilibration in either N_2_(*g*), 21% O_2_(g) or N_2_(g) in the presence of 50 mM nickel ethylenediamine‐N,N′‐diacetic acid (Ni‐EDDA). Samples loaded into TPX capillary tubes were purged with N_2_(*g*) inside the EPR resonator for 30 min prior to each experiment. Experimental conditions were as above except the microwave power was varied in 3 dB steps (from 0.001 to 50 mW) and the number of scans accumulated per power (in normalised acquisition mode) was varied until a reasonable signal‐to‐noise ratio per spectrum was achieved.

The microwave power required to reduce the vertical peak‐to‐peak amplitude of the central ($M_{i}=0$) resonance of the first derivative EPR spectrum of MTSL (for e.g. see Figure 4 and Figures S9–10) to 50% of its unsaturated value is known as $P_{½}$ and is proportional to the longitudinal relaxation rate of the nitroxide label. A $P_{½}\left(\text{in}\;N_{2}\right)$ describes the saturation profile in the absence of any paramagnetic quenchers whereas any collisions with paramagnetic relaxing agents such as O_2_ and Ni‐EDDA may increase the power required to saturate the EPR signal to 50% that is, $P_{½}\left(O_{2}\right)\&P_{½}\left(\mathrm{Ni}-\text{EDDA}\right)$. An accessibility parameter, Π, calculated as described previously, measures the individual collision frequency of a nitroxide label with any paramagnetic quencher. This parameter should be normalised against a known reference (here 2,2‐diphenyl‐1‐picrylhydrazyl, DPPH) and mean Π values calculated for each conformational configuration of LeuT K398C. A polarity index or depth parameter, Φ, can also be determined for each LeuT K398C conformational state in proteoliposomes as described previously (Altenbach et al. 1994; Altenbach et al. 1989; Altenbach et al. 1990; Nielsen et al. 2005; Malmberg and Falke 2005). Generally, a positive Φ value describes a situation where the spin label is ‘buried’ within a more hydrophobic lipid environment, typical of a biological membrane where only collisions with molecular oxygen can occur, whereas a negative Φ value represents a spin label exposed to a more aqueous phase, for example, on a membrane surface or in exposed TM regions where Ni‐EDDA‐dependent collisions can dominate. All power saturation data were fitted using MATLAB utilising the standard power saturation expression (see Supporting Information—Data S1; (van Wonderen et al. 2014; Hemminga and Berliner 2007)).

### 2.6 *In Silico* Site‐Directed Spin Labelling and Protein Structure Visualisation

MTSL rotamer libraries at positions K398 and R86 were generated by the open‐source MATLAB‐based molecular modelling toolbox, Multiscale Modelling of Macromolecular systems (MMM version 2018.2) (Jeschke 2018). Rotamer ensembles were calculated at ambient temperature (298 K) using LeuT crystal structures depicting the different conformational states: *OOp* absence of substrate and presence of Na^+^ ions (PDB: 3TT1), *OOpW* in the presence of an inhibitor (tryptophan) and Na^+^ ions (PDB: 3F3A), *OOcL* (PDB: 2A65) state in the presence of both substrate (leucine) and Na^+^ ions, *OOcA* (PDB: 3F48) state in the presence of both substrate (alanine) and Na^+^ ions, *OOCF* (PDB: 6XWM) in the presence of phenylalanine and Na^+^ ions, *IOpApo* in the absence of both Na^+^ ions and substrate (PDB: 3TT3), and *OReApo* state in the absence of both Na^+^ ions and substrate (PDB: 5JAE). A rotamer analysis provides a statistical distribution of spin label side chain conformations attached at positions K398C and R86C. Rotamer libraries were visualised, processed, and rendered using “UCSF Chimera” (v. 1.16, https://www.rbvi.ucsf.edu/chimera/). LeuT structures without *in silico* attached MTSL rotamer libraries were visualised using “UCSF ChimeraX” (v. 1.7.1, https://www.rbvi.ucsf.edu/chimerax/) (Pettersen et al. 2021; Pettersen et al. 2004).

### 2.7 EPR Spectral Simulations

All cw EPR spectra were simulated using the MATLAB‐based toolbox EasySpin (Stoll and Schweiger 2006). For EPR data at ambient temperature (~293 K) the ‘chilli’ function for the slow‐motion regime was used, while data obtained at cryogenic temperatures (50 K) were analysed with the ‘pepper’ function, equivalent to the rigid, motional limit of nitroxides, to give accurate EPR parameters including the g‐tensor elements ($\hat{g}$), hyperfine coupling constants (*a*), rotational correlation times (*τ*_*c*_), as well as the relative weightings for multi‐component data.

## 3 Results

### 3.1 Purification and Efficient Spin Labeling of LeuT K398C and R86C With MTSL

Recombinant LeuT cysteine mutants, K398C and R86C, were purified via a novel single IMAC/SDSL step during the purification procedure, resulting in higher (~60%) spin‐labelling efficiency (see Supporting Information—Data S1 and Figure S1 for details).

### 3.2 Computational EPR Spin Labelling of K398C and R86C Based on a Rotamer Library Approach

The three‐dimensional space occupied by each rotamer ensemble attached at position 398 in five key LeuT conformations is depicted in Figures S7 and S8, while Table S3 describes the specific number of MTSL rotamer populations for all known conformations (respective images are shown in Figure S8). The MTSL rotamer populations for K398C are increased in all outward‐facing conformations and decreased in all inward‐facing conformations, whereas for R86C they remain relatively constant.

### 3.3 Continuous‐Wave EPR of K398C in Detergent Micelles and Proteoliposomes

**FIGURE 2:**
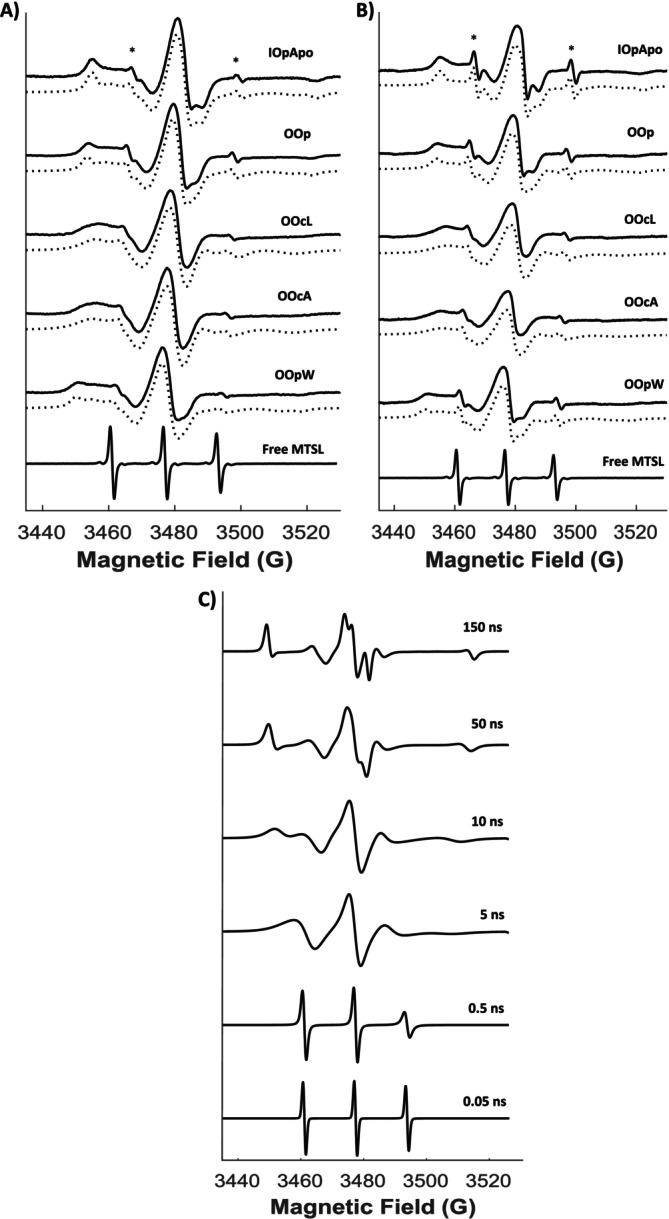
(A) X‐band cw EPR spectra of MTSL bound to LeuT K398C in detergent‐solubilised DDM micelles under the distinct ion/ligand conformational states: *IOpApo, OOp, OOcL*, *OOcA*, *OOpW* (from top to bottom) and unattached in buffer (lowest trace). A small contribution of unspecific free label (<~2%–5%) remains in all protein‐bound sample spectra (*). EPR spectral simulations are shown below each experimental spectrum (dotted lines). (B) X‐band cw EPR spectra of MTSL bound to LeuT K398C in reconstituted proteoliposomes (PL) as in A. (C) Representative, theoretical X‐band cw EPR spectra of a nitroxide spin label with different side chain motions described by a rotational correlation time (*τ*_*c*_) in ns, generated using EasySpin in MATLAB (Stoll and Schweiger 2006). *IOpApo*, inward facing open apo, *OOp*, outward facing open (Na^+^‐bound); *OOcL*, outward facing occluded with leucine (Na^+^/leucine‐bound); *OOcA*, outward facing occluded with alanine (Na^+^/alanine‐bound), *OOpW*, outward facing open with tryptophan (Na^+^/tryptophan‐bound).

Continuous wave (cw‐) EPR spectra of spin labelled K398C at ambient temperature in the *IOpApo*, *OOp*, *OOcL*, *OOcA* and *OOpW* states in both detergent‐solubilised DDM micelles (Figure 2A) and proteoliposomes (Figure 2B) are shown together with free MTSL in solution for comparison (lowest trace). Comparable data for R86C are shown in Figure S3. The overall spectral appearance of all LeuT‐bound EPR spectra is much broader when compared to the three sharp peaks seen for an unattached, mobile MTSL label in solution. These sharp peaks arise from hyperfine coupling of the unpaired electron with a single ^14^N nucleus ($m_{I}=1$) from the nitroxide radical (NO^•^) which is free to tumble rapidly in solution (lowest trace, Figure 2A,B).

**FIGURE 3:**
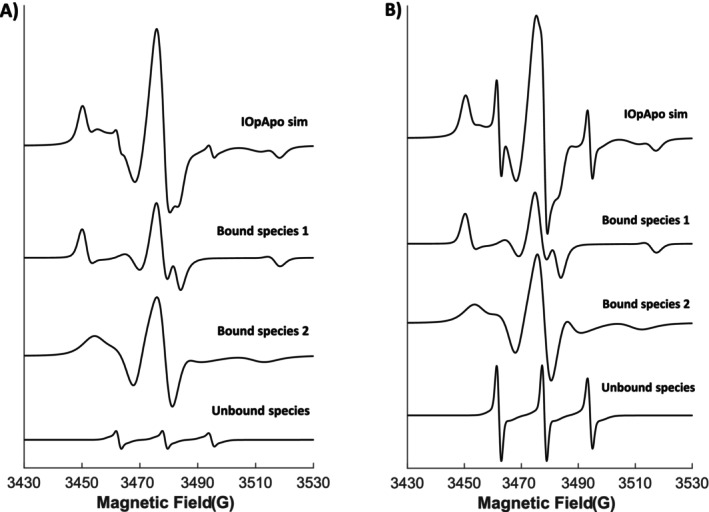
Representation of the individual cw spectral components for K398C *IOpApo* conformation in (A) detergent‐solubilised (DDM) micelles and (B) reconstituted proteoliposomes with the combined simulation output comprising the same spectra as presented in Figure 2A,B (dotted lines), respectively, and generated by the MATLAB–based EasySpin toolbox. The intensities of the individual spectra of bound species are given in Table 1; the contribution of unbound species is ~2%–6%.

The spectral broadness observed in Figure 2A is characteristic of highly restricted motions of a protein‐bound spin label which can be described by a rotational correlation time (*τ*_*c*_) whose magnitude typically falls within the nanosecond (ns, slow tumbling) to microsecond (μs, fully immobilised or frozen) time scale and which dominates the overall appearance of the observed EPR line shape. For LeuT K398C EPR spectra recorded in different conformations, the observed EPR line shapes are rather complex with a very slight, but consistent (~2%–4%) component which is assigned to unspecific free MTSL label (indicated with * in Figure 2A,B and the “unbound” species as individual spectral components in Figure 3 and Figure S6). Spectral simulations using the full spin Hamiltonian parameters including especially $\tau_{c}$ values permit a distinction between slow tumbling nitroxides buried within a protein or membrane (> 4 ns) and labels with high‐motional freedom exposed on protein surfaces (< 1 ns) (Figure 2C) (Mobius et al. 2005).

The complex line shape associated with the protein‐bound label varies depending on which conformation is present. In detergent micelles, the *IOpApo* state reveals via spectra simulation two distinct spin‐label rotameric conformations present in a 2:1 ratio with correlation times (*τ*_*c*_) of 7 ns and 50 ns respectively (see Table 1, Figure 3, and Table S2). Incubation with Na^+^ results in formation of *OOp*, where this ratio shifts towards 3:1, and only upon incubation with leucine or alanine to form *OOcL* or *OOcA* is a single rotameric conformation (*τ*_*c*_ of 7 ns) observed. Interestingly, incubation with tryptophan to form *OOpW* does not result in the formation of a single rotameric conformation but rather appears to shift the ratio towards 4:1. In proteoliposomes, similar trends are observed except that the ratio is already 4:1 in *OOp* and does not alter further upon incubation to form *OOpW*, except that the *τ*_*c*_'s in these two states are slightly altered to 106 ns/4 ns respectively, indicating both a more buried and more exposed position of the label with respect to that in detergent micelles. In addition, all individual spectral components for the cw EPR dataset depicting the *IOpApo* conformational state in both detergent‐solubilised micelles (Figure 3A) and reconstituted proteoliposomes (Figure 3B) are presented, while similar spectral analyses for the other ion/substrate‐induced conformations (*OOp*, *OOcL*, *OOcA*, *OOpW*) can be found in the Supporting Information (Figure S6).

**TABLE 1:** Characterization of conformations of EPR rotamer populations of LeuT K398C.

State	Detergent micelles (DM)				Proteoliposomes (PL)			
	First population		Second population		First population		Second population	
	Fraction	*τ*_*c*_/ns	Fraction	*τ*_*c*_/ns	Fraction	*τ*_*c*_/ns	Fraction	*τ*_*c*_/ns
*IOpApo*	0.64	6.9	0.34	50.2	0.60	6.5	0.34	56.0
*OOp*	0.71	7.1	0.24	48.8	0.82	4.1	0.16	106.3
*OOcL*	0.94	7.6	—	—	0.96	6.0	—	—
*OOcA*	0.95	6.8	—	—	0.95	5.1	—	—
*OOpW*	0.81	11.1	0.16	47.5	0.82	4.9	0.16	67.0

Abbreviations: IOpApo, inward facing open apo; OOcA, outward facing occluded with alanine (Na^+^/alanine‐bound); OOcL, outward facing occluded with leucine (Na^+^/leucine‐bound); OOp, outward facing open (Na^+^‐bound); OOpW, outward facing open with tryptophan (Na^+^/tryptophan‐bound).

### 3.4 Cw EPR Power Saturation of K398C in Detergent Micelles and Proteoliposomes

**FIGURE 4:**
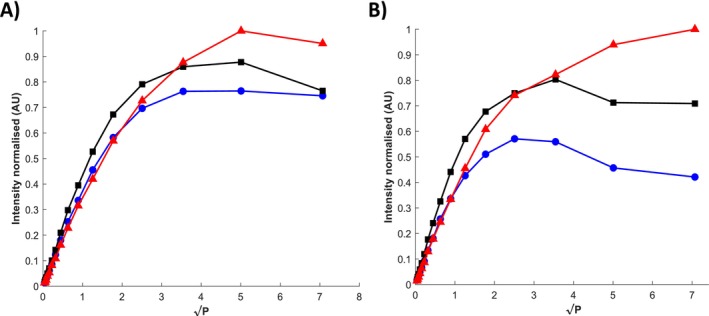
Normalised cw EPR power saturation curves of LeuT K398C in detergent‐solubilised micelles (A) and proteoliposomes (B) under the *IOpApo* conformation. The cw EPR spectra used to generate the saturation profiles were recorded in the presence of O_2_ (blue, circles), N_2_ (black, squares), and 50 mM Ni‐EDDA (red, triangles). Details of the data analysis are given in the Supporting Information—Data S1. *IOpApo*, inward facing open Apo.

The cw EPR power saturation profiles of spin‐labeled K398C in the *IOpApo* state are shown in Figure 4. Clear differences in the saturation profiles are observed in the presence of both paramagnetic quenchers (O_2_, Ni‐EDDA) as compared to diamagnetic N_2_. Similar data have been recorded for all the other conformational configurations studied (*OOp*, *OOcL*, *OOcA*, *OOpW*) in both detergent micelles and proteoliposomes (presented in Figure S9). Analysis of EPR power saturation was performed as described in the materials and methods using the equations presented in the Supporting Information and is given in Table 2. Solvent exposure of cysteine‐bound nitroxide spin labels can be estimated by determining the collision frequency with either water‐soluble paramagnetic relaxants (e.g., Ni‐EDDA) or non‐polar paramagnetic relaxation agents (O_2_). Molecular oxygen (O_2_) is imperfectly soluble in aqueous surfaces but is highly concentrated within the hydrophobic cores of membrane proteins and lipid bilayers. Conversely, Ni‐EDDA is easily accessible to solvent phases (including regions within solvent‐accessible cavities and pores in a membrane protein), but not those ‘buried’ in transmembrane cores or directly exposed to the lipid bilayer. Therefore, Ni‐EDDA accessibility decreases within a lipid bilayer's hydrophobic environment, while O_2_ accessibility increases in a complementary manner.

**TABLE 2:** EPR accessibility profiles of spin‐labelled LeuT K398C in detergent‐solubilised micelles (*top*) and reconstituted proteoliposomes (*bottom*).

Detergent micelles (DM)					
State	P_½_ (mW) (O_2_)	P_½_ (mW) (Ni‐EDDA)	Accessibility Π (O_2_)	Accessibility Π (Ni‐EDDA)	Polarity index (Φ)
*IOpApo*	10.2	15.9	0.021	0.890	−3.765
*OOp*	13.9	27.3	0.465	2.461	−1.667
*OOcL*	21.1	20.0	0.425	0.248	0.540
*OOcA*	24.2	22.4	1.694	1.282	0.279
*OOpW*	34.9	16.5	2.595	0.057	3.825

Proteoliposomes (PL)					
*IOpApo*	4.3	5.9	0.017	0.252	−2.670
*OOp*	7.2	5.1	0.445	0.091	1.581
*OOcL*	16.8	8.8	1.298	0.108	2.486
*OOcA*	5.4	4.9	0.086	0.002	4.013
*OOpW*	7.1	6.4	0.176	0.077	0.830

Abbreviations: IOpApo, inward facing open apo; OOcA, outward facing occluded with alanine (Na^+^/alanine‐bound); OOcL, outward facing occluded with leucine (Na^+^/leucine‐bound); OOp, outward facing open (Na^+^‐bound); OOpW, outward facing open with tryptophan (Na^+^/tryptophan‐bound).

A comparison of the accessibility parameters Π (O_2_) and Π (Ni‐EDDA) in the ion/substrate‐free conformation (*IOpApo*) indicate that the attached nitroxide label at position K398C has a much greater accessibility to Ni‐EDDA in both detergent micelles and proteoliposomes, indicative of a residue that is likely exposed to the aqueous phase in both environments. In comparison, Π (O_2_) remains relatively constant between both environments while Π (Ni‐EDDA) is significantly decreased in proteoliposomes indicative of a spin label whose collisional access to Ni‐EDDA is more perturbed by the presence of lipids. These observations are further supported by comparison of the polarity indexes (or membrane depth parameter), Φ, between the two environments which indicate that the label at K398C is highly exposed to a polar, aqueous environment in both states in the *IOpApo* conformation although exposure is much greater in detergent micelles than in proteoliposomes.

Interestingly, in the *OOp* conformation, a ~3‐fold increase in Π (Ni‐EDDA) and ~22‐fold increase in Π (O_2_) is observed compared to the *IOpApo* state in detergent micelles indicating that the label has transitioned towards a more dynamic state allowing motion between the aqueous and lipid phase. In contrast, in proteoliposomes a similar large increase in Π (O_2_) is observed but a ~3‐fold decrease in Π (Ni‐EDDA) which indicates a more buried position of the label in the lipid environment where only O_2_ is accessible and expected to have a large effect. Again, this is also reflected in the Φ values between the two environments where the label remains particularly exposed to the polar solvent in detergent micelles but now appears submerged within hydrophobic lipids in proteoliposomes. Overall, a direct comparison of the polarity indexes between the *IOpApo* and *OOp* conformations in both environments ($\left|∆\Phi_{\mathrm{DM}}\right|=2.1$, $\left|∆\Phi_{\mathrm{PL}}\right|=4.25$) suggest that K398C faces a more hydrophobic environment in the *OOp* conformation in proteoliposomes than in detergent micelles.

After subsequent addition of leucine, and formation of the *OOcL* configuration, Π (O_2_) remains relatively constant while Π (Ni‐EDDA) is markedly decreased (~10‐fold) in detergent micelles suggesting that the label is now in a more buried, inaccessible position within the protein interior. In contrast, in proteoliposomes, a considerable increase in Π (O_2_) is seen while Π (Ni‐EDDA) remains constant and low suggesting that the label has moved deeper into the lipid bilayer after substrate*‐dependent* conformational rearrangement. Overall, after leucine binding, the label at K398C is found in a more hydrophobic domain in both environments, as also seen by the Φ values, compared to the *OOp* state.

For substrate‐binding using alanine to form the *OOcA* conformation the greatest differences are observed between detergent micelles and proteoliposomes. Both Π (Ο_2_) and Π (Ni‐EDDA) are significantly increased in detergent micelles as compared to the *OOcL* conformation and the polarity index is slightly reduced demonstrating a comparatively more solvent‐exposed position of K398C. Such a simultaneous increase of both Π (Ο_2_) (~4‐fold) and Π (Ni‐EDDA) (~5‐fold) may reflect a similar situation as seen above for the *OOp* state in detergent micelles with highly dynamic label fluctuations at the protein surface. Surprisingly however, both Π (Ο_2_) and Π (Ni‐EDDA) are dramatically reduced ~15‐fold and ~54‐fold, respectively, in proteoliposomes, which can only be reconciled with the label being significantly buried deeper within the protein interior caused by the presence of the liposome surrounding. A closer inspection of the polarity index (Φ) highlights a slightly more polar surrounding in detergent micelles compared to the *OOcL* state ($\Phi_{A}=0.28$, $\Phi_{L}=0.54$, $\Phi_{A}/\Phi_{L}\sim 0.5-\text{fold}$) while in proteoliposomes the label faces the most hydrophobic environment ($\Phi_{A}=4.01$), which is further supported by the lowest Π (Ni‐EDDA) reported under all induced conformational states in this study.

Finally, the competitive inhibitor, tryptophan (Singh et al. 2008) was used to induce a *OOpW* conformational state and resulted in the highest Π (O_2_) and lowest Π (Ni‐EDDA) compared to all conformational states in detergent micelles which is unusual. This could be explained if the label is mostly embedded within a hydrophobic region of the protein but is not buried in the protein interior or a solvent‐accessible cleft, an observation that immediately contradicts the EPR accessibility parameters obtained for the *OOp* state and the *OOp* conformation seen in the *inhibitor‐bound* crystal structure (PDB: 3F3A) (Singh et al. 2008). The shifts in Π (Ο_2_) (~2.5‐fold and ~7.4‐fold decreases respectively), and Π (Ni‐EDDA) (~1.2‐fold and ~ 1.4‐fold decreases), respectively compared to the *OOp* and *OOcL* conformations in proteoliposomes (Singh et al. 2008) can be explained by a label which is found in a similar or a slightly more polar environment, but is displaced from the hydrophobic, lipid bilayer, supported by the low Φ value ($\Phi_{W}=0.83$). This contrasted with the high Φ value observed in detergent micelles ($\Phi_{W}=3.825$) and the variance in this accessibility data clearly reflects the different positions of the residue in the extracellular region at the membrane lipid interface in this conformation seen in both environments.

Comparable cw EPR power saturation profiles for spin labelled R86C in all possible states remain quite constant (see Table S4) and do not display the variations seen for K398C during transport (see Figure S10 for a direct comparison of the power saturation profiles between the two LeuT cysteine mutants, K398C and R86C).

## 4 Discussion

### 4.1 Why Was Position K398 Chosen to Study?

Currently, TM10 is considered part of LeuT's more stable ‘scaffold’ domain comprising of TMs 3–5 and 8–10 while the ‘bundle’ domain contains TMs 1–2 and 6–7 and moves relative to the former with a proposed ‘rocking‐bundle’ transport mechanism. After substrate binding, TM1 moves closer to TM10 and creates an extracellular gate that seals access from the extracellular milieu to the S1 binding site by forming a salt bridge between Arg‐30 (R30, TM1b) and Asp‐404 (D404, TM10) (Yamashita et al. 2005; Singh et al. 2007). In addition, Ala‐319 on extracellular loop 4b (EL4b) comes into closer proximity to Asp‐401 on TM10 and in combination with the R30‐D404 salt bridge results in the complete closure of the extracellular permeation pathway towards the substrate binding site. Previous studies have postulated slight movements in TM10 (Claxton et al. 2010; Calugareanu et al. 2022), but no global, coordinated alteration has so far been observed (Thomas et al. 2014). Using SDSL and EPR spectroscopy, the aim was to investigate the subtle dynamics of the extracellular region of TM10 in more depth and its specific role in LeuT's transport cycle, further enhancing (or challenging) current static information available from the crystal structures.

### 4.2 Spin‐Labelling Suitability of Residue K398 and Interpretation of *In Silico* EPR Analyses

**FIGURE 5:**
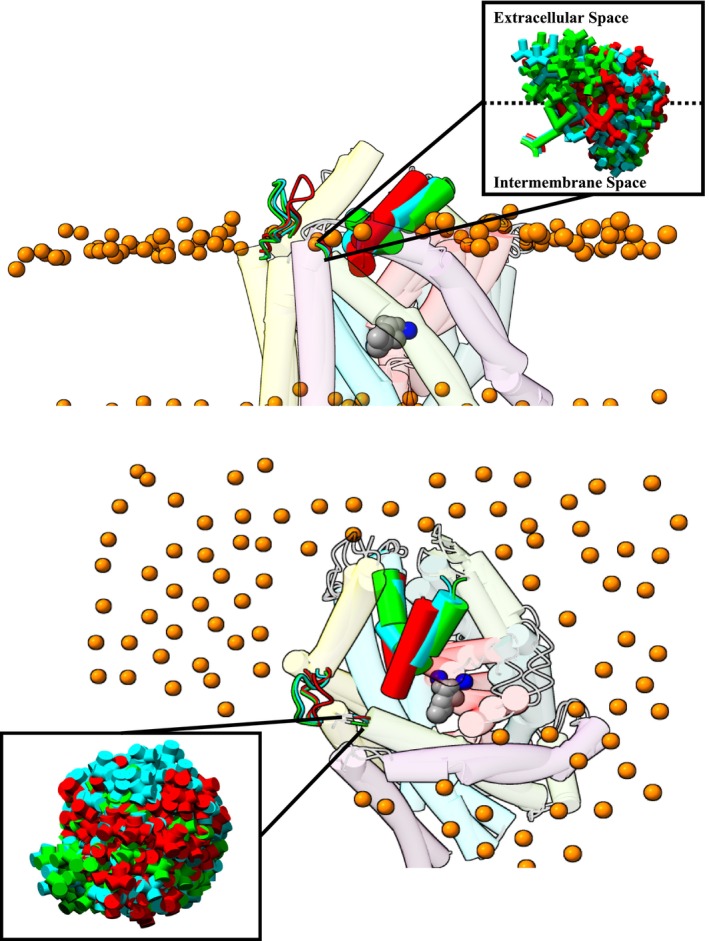
Visualisation from the side perspective (top) and from above the membrane (bottom) of the local environment of the attached MTSL rotamers on K398C. K398 is located on the lipid membrane‐solvent interface at the extracellular part of the transporter. Similar to Figure 1, the predicted lipid bilayer plane is highlighted by phosphorus atoms (*orange spheres*) of the phospholipids' head group together with the two sodium ions (*blue spheres*) and the substrate L‐leucine (*grey spheres*) while the rest of the LeuT backbone is set to transparent for clarity. The connecting loop between EL2 and TM4, residue K398 and EL4 are highlighted in the *OOp* state (*3TT1, green*), *OOcL* state (*2A65, cyan*), and *IOpApo* state (*3TT3, red*). All MTSL rotamers in these conformational configurations are visualised as sticks (in respective colours) and populate both the extracellular milieu and intermembrane space (box zoomed on the rotamers). All MTSL rotamers were computationally attached using MMM while visualisation of all structural models was performed on ‘UCSF’ Chimera and ‘UCSF’ ChimeraX.

A computational approach, using MMM, was able to calculate, computationally attach, and visualize an MTSL rotamer library at position K398 (using variant K398C) at ambient temperature (298 K) under all conformational states by using existing LeuT crystal structures as reference models. K398 is located on the extracellular part of a connecting region between TM10 and TM9 and resides at the lipid membrane‐polar solvent interface of the transporter (Figures 1 and 5).

The large number of MTSL rotamers (Table S3) per conformational configuration confirms the suitability of K398 for successful spin labeling and study by EPR. The large number of rotamers suggests an accessible labeling site with on average ~58 MTSL molecule positions in all outward‐facing conformations, which contrasts with an average of only ~35 for all inward‐facing structures. This 0.6‐fold decrease in the available number of attached MTSL rotamers in the inward‐facing conformations is expected and highlights potential subtle changes of TM10 during the closure of the extracellular permeation pathway after substrate binding. For residue R86C, the number of rotamers remains constant (between 133 and 141) for both inward and outward‐facing conformations, further supporting R86C as a suitable static control. It should, however, be noted that these crystal structures only depict a static snapshot of the overall transport cycle and may not fully describe the entire flexibility and coordinated movements occurring in the transporter in response to the binding of ions and substrate. Finally, since the *in silico* MMM analysis was performed using different LeuT crystal models, it is imperative to recognise that not all of them describe a wild‐type equivalent, but rather a series of mutational studies which were able to ‘lock’ LeuT into specific preferred conformations prior to crystallisation. Mutations are present in the following PDB entries: 3F3A (*OOpW*), 6XWM (*IOcF*), and 3TT3 (*IOpApo*). Although useful mechanistic information has previously been obtained from these crystal structures, these mutations may alter LeuT's overall tertiary structure, potentially affecting all downstream *in silico a*nalyses as well as the interpretation of experimental measurements (Krishnamurthy and Gouaux 2012; Gotfryd et al. 2020). In Figure 5, the distribution of rotamers in *IOpApo* (red) reveals two regions of rotamer ‘*density*’ above and below the membrane interface, while for the *OOp* state (green) this *density* now appears slightly more spherical and more exposed above the membrane interface. Finally, the *OOcL* state (cyan) rotamer *density* is again much more spherical but now more buried beneath the membrane interface.

### 4.3 Assigning and Quantifying Conformational Ensembles on LeuT in Detergent Solubilised Micelles and Proteoliposomes Using Cw EPR Spectral Analyses of Spin Labelled K398C

Previous experimental and computational approaches that attempted to assign LeuT's *IOpApo* configuration generally suggested that the transporter was in an equilibrium comprised of both closed and open intermediate conformations. This was first described through the observation of a population mixture of short‐ and long‐distance distributions obtained from a double cysteine variant H480C/A309C using DEER spectroscopy (Claxton et al. 2010), a phenomenon that had also been reported for the *apo* state in the Na^+^‐coupled hydantoin symporter, Mhp1, that utilises a similar LeuT‐fold topology (Li et al. 2019; Kazmier et al. 2014b). Spectral analysis of the cw EPR data of K398C in the *IOpApo* conformational state also indicates that the attached spin label can adopt two slightly different environments, in close agreement with previous studies, although based on this work the EPR line shape seen in the earlier work is indicative of incomplete substrate binding in the *OOcL* state. This is further supported by single‐molecule FRET (smFRET) experiments which propose that the extracellular and intracellular sides of LeuT are not tightly coupled, as described during the alternating access transport mechanism, but rather that the transporter is able to fluctuate between *apo*, *OOp*, and *IOp* states (Terry et al. 2018). In contrast, based on the findings of a hydrogen/deuterium exchange mass spectrometry (HDX‐MS) study, it was hypothesised that under *apo* conditions LeuT primarily adopts an *OOp* conformation, which is further stabilised energetically by the binding of two Na^+^ ions (Merkle et al. 2018). Note that the crystal structure representing LeuT stabilised in an *IOpApo* conformation (PDB: 3TT3) could only be obtained after conducting a series of mutations on TM3 (scaffold domain), TM6 (bundle domain) and TM8 (scaffold domain), with the latter helix also containing mutations further destabilising the Na_2_ binding site (Krishnamurthy and Gouaux 2012). Taking the above into consideration, currently the *IOp* conformation of LeuT does not seem to represent a wild‐type structure and/or functional equivalent, and it is proposed that the term ‘*apo*’ should be used with caution.

It was previously demonstrated that potassium (K^+^) ions play a pivotal role in regulating the LeuT transport cycle, and hence possibly also NSS proteins, by competitively inhibiting both the rebinding of Na^+^ ions as well as the Na^+^‐dependent rebinding of substrate in the inward‐facing open conformation (Billesbolle et al. 2016; Schmidt et al. 2024). The *IOpApo* state studied here is in the absence of Na^+^ but is in the presence of K^+^ ions (200 mM KCl) that serve as a ‘control’ conformational state to generate the other conformations, *OOp*, *OOcL*/*OOcA*, and *OOpW* states studied (see Materials and Methods). Therefore, any potential effect of K^+^ binding in the *IOpApo* state shifting LeuT towards a K^+^‐induced, outward‐closed/inward‐open conformation needs to be considered. EPR data recorded in the *IOpApo* configuration reveal a strongly immobilized nitroxide label reflecting restricted motion in a buried local environment, which fits with an outward‐closed/inward‐open conformation stabilized by concurrent binding of K^+^ ions in the intracellular gate in strong agreement with the recently reported literature (Billesbolle et al. 2016; Schmidt et al. 2024). Note, however, that since the *IOpApo* configuration may also comprise an ensemble of *OOp* (*τ*_*c*_ ~ 6.87 ns) and *IOp* (*τ*_*c*_ ~ 50.2 ns) states and describes two unequal EPR spectral populations (see Figure 3 and Table 1), which proposed that this was slightly shifted towards an *OOp* conformation under the given experimental conditions. LeuT adopts these preferred configurations simultaneously in both detergent micelles and proteoliposomes in a ~ 2:1 ratio. Previous single‐molecule fluorescence studies have also described similar conformational ensembles often suppressed under crystallographic conditions (Zhao et al. 2010). Another recent study utilized 1D ^19^F–NMR spectroscopy on LeuT K398C for the first time in an attempt to assign conformational states on the transporter based on detectable, global, structural conformational rearrangements on the extracellular permeation pathway in detergent—solubilised micelles (Daminato et al. 2025). It was observed that K^+^ ions interact with the transporter and generate a shift in the conformational ensembles towards inward–facing intermediates. Thus, all experimental results derived from a combination of several spectroscopic, biophysical methods thus far (EPR, NMR and tmFRET spectroscopy) further reinforce the hypothesis that K^+^ ions can affect/regulate the transport cycle of LeuT, an effect that seems to be consistent across different environments (detergent micelles and proteoliposomes). Additionally, both EPR and 1D ^19^F–NMR spectroscopic data could not deconvolute between inward–facing open and inward–facing closed conformations.

Addition of Na^+^ ions counteracts this K^+^‐induced outward‐closed/inward‐open conformation and further stabilises LeuT in an *OOp* state, which is directly reflected in the shift of the EPR spectral component ratio towards more mobile spin populations with a 3:1 and 4:1 ratio in detergent‐solubilised micelles and reconstituted proteoliposomes, respectively (see Figures 2 and 3; Figure S6; Table 1 and Table S2). After Na^+^‐dependent binding of leucine and alanine to form the *OOcL*/*OOcA* states, a clear shift in populations as compared to the *OOp* configuration occurred with only one dominant, mobile spectral component present in both membrane environments (see Figure 2; Figure S6; Table 1 and Table S2). It can be hypothesised that since LeuT is only embedded in detergent micelles, there will be no directionality of a Na^+^ gradient, as would be seen in a native lipid bilayer, to promote the transport of substrate; thus, the Na^+^‐dependent binding of leucine or alanine alone is able to switch LeuT from an *OOp* to the more thermodynamically stable *OOc* position, but may not induce fully additional global structural changes underlying a more *IOc* rearrangement, since the ratios observed by EPR shift more in proteoliposomes. Both the ratios of species detected by EPR and the distribution of their rotamers seen by MMM agree well. Both the *OOp* (*3TT1*, green) and *OOc* (*2A65*, cyan) LeuT rotameric distributions are much more isotropic and less significantly altered than the *IOpApo* conformation (3TT3, red) (Figure 5). Experimental evidence from smFRET indicated that leucine, as a bulkier amino acid, is poorly transported towards the S1 binding site, while the smaller alanine can bind and be transported more efficiently by LeuT (Terry et al. 2018). In addition, it was shown that substrate binding contributes to the closure of the extracellular pathway by destabilising the transporter's *OOp* open conformation, which supports the observations made here of change in spin label accessibility (Table 2). The addition of tryptophan to a Na^+^‐induced conformational state on LeuT and the formation of the *OOpW* inhibited conformation showed the same 4:1 ratio of spectral components in both environments (see Figure 2, Figure S6; Table 1 and Table S2) with the local environment of the attached spin label resembling the *OOp* state. It can be speculated that this observation from the EPR experiments represents and verifies the inhibitory effect on LeuT, which traps the transporter into an outward‐open configuration.

To summarise, by combining the information obtained from cw EPR spectral analyses and simulations of K398C, it was observed that the *IOpApo* state of LeuT is a mixture of conformational ensembles that are reflected in the distinct spin‐label populations seen in the EPR spectral simulations (Table 1). Additionally, the population ratio in the *IOpApo* conformation was determined to be ~2:1, with both the mobile and immobilised components corresponding to the respective *OOp* and the *IOp* configurations for both membrane environments. Furthermore, after the addition of Na^+^ ions, a shift in the rotameric populations to ~3:1 and ~ 4:1 was observed for both the detergent‐solubilised micelles and reconstituted proteoliposomes membrane environments, respectively. The previous observation aligns with the current understanding of the transport cycle of LeuT, where Na^+^ ions stabilise the *OOp* conformation, further validated by the increased mobile component in both membrane environments. However, the immobilised component observed in the *OOp* configuration could be justified by a fraction of LeuT molecules not being able to completely adopt the outward‐open state due to the existing effect of K^+^ ions (see above). After the addition of either leucine or alanine to the Na^+^‐induced conformation (*OOp*) a dramatic shift towards a single mobile component was observed in the respective *OOcL* and *OOcA* conformations across both membrane environments. This demonstrates that K398 and, subsequently, the extracellular domain of TM10 is more dynamic compared to the in silico EPR analyses performed on the ‘static’ crystallographic structures, which show minimal differences in rotameric distributions between the *OOp* and *OOcL/OOcA* configurations (see Figures S7 and S8; Table S3). Similarly, after the addition of the inhibitor tryptophan and the entrapment of the transporter in an outward‐open inhibited state (*OOpW*), the shift in the rotameric populations towards a ~4:1 ratio in both membrane environments aligns with our observations on the *OOp* configuration.

### 4.4 The Spin Label Accessibility Profiles of K398C in Detergent Solubilised Micelles and Proteoliposomes Exhibit Both Similarities and Divergences From the LeuT Crystal Models

In the only previous LeuT EPR accessibility study (Claxton et al. 2010), residues N397C, L400C, D404C, and F405C in TM10 were investigated in reconstituted proteoliposomes under different ion/substrate binding conformations. N397C revealed the highest Π (Ni‐EDDA) in the *OOp* conformation whereas no significant differences were observed between the *IOpApo* and *OOp* states for the more buried L400C and D404C positions. In the *OOcL* conformation, however, all positions exhibited a profound decrease in Π (Ni‐EDDA) suggesting a substrate‐dependent closure of the extracellular vestibule where LeuT adopts an *OOc* state by blocking solvent access to the S1 binding site through the extracellular permeation pathway (vestibule), very similar to another LeuT‐fold transporter, Mhp1 (Weyand et al. 2008). Overall, K398C EPR accessibility data agrees well with this proposal in both detergent micelles and proteoliposomes (Table 2).

Here, however, a further interesting phenomenon is observed, a decreased Π (Ni‐EDDA) between the *IOpApo* and *OOp* conformational states in proteoliposomes, which was not reported previously by Claxton et al. that indicates that K398 is more exposed to the solvent in the *IOpApo* state only in proteoliposomes. Since K398C is located close to the lipid membrane‐polar solvent interface, this may be attributed to the different nature of the encapsulating surrounding of LeuT between detergent‐solubilised micelles and proteoliposomes and could potentially have an impact on the three‐dimensional topology of the embedded LeuT (Mondal et al. 2013; Sohail et al. 2016). Further, quantification of the EPR spectral simulations for the *IOpApo* state indicates that a mixture of conformational ensembles co‐exist presumably between *OOp* and *IOp* configurations.

Successive substrate binding results in the formation of the *OOcA*/*OOcL* conformational state where K398C is found in a more buried environment in both detergent micelles and proteoliposomes compared to the *IOpApo* and *OOp* states, which agrees well with the earlier interpretations of Claxton et al. and is explained by the closure of the EV. Interestingly, it is speculated that the higher hydrophobicity of K398C in *OOcA*/*OOcL* configurations in proteoliposomes compared to detergent solubilised micelles occurs due to the presence of the Na^+^ gradient that may allow the transporter to fully adopt ion/substrate‐dependent occluded configurations, which is reflected in the dynamic movement of the extracellular part of TM10 (K398C) towards the protein interior and lipid bilayer to seal the extracellular permeation pathway. As well as forming part of the ‘scaffold’ domain, TM10 has also been termed a ‘gating helix’ since it is responsible for regulating the opening/closure of the extracellular cavity by bending towards and interacting with TM1 to occlude the S1 primary binding site. Direct comparison of TMs 1 and 10 suggests a simultaneous concerted movement of both helices towards S1 between the *OOp* (green), *OOc* (cyan), and *IOp* (red) conformations (Figure S7) in the crystal structures. Specifically, TM1a reveals a significant structural rearrangement in the *IOp* conformation only that contributes to an opening of the intracellular pathway, while TM1b closes the extracellular gate in the *OOc* (cyan) and *IOp* (red) conformational states as compared to the *OOp* configuration (green). TM10 also exhibits a more subtle tilt towards TM1b after Na^+^‐dependent substrate occlusion, which results in the formation of a salt bridge (Arg30‐Asp404) to close the extracellular gate and which is retained in the *IOp* conformation (Krishnamurthy and Gouaux 2012). The EPR accessibility data reported here for K398C, which is located between the extracellular end of TM10 and the lipid membrane interface, strongly support this movement of TM10 in agreement with both previous EPR studies (Claxton et al. 2010) that had hinted at such a movement and with current static crystal structure models. Additionally, tmFRET measurements between a large, bulky fluorescent probe attached at K398C (K398C^FL^) and a divalent metal ion coordinated by a genetically engineered double‐histidine motif (A313H‐A317H) on extracellular loop 4 (EL4) have also provided further experimental evidence for a more closed conformation after substrate binding as compared to static crystal structure models, again supporting the hydrophobicity seen in the EPR accessibility data. Thus, in addition to the label's interaction with the membrane interface, its local environment hydrophobicity also arises from its proximity to EL4 which influences its EPR saturation behaviour and its accessibility towards paramagnetic quenchers. Despite the subtle conformational changes reported for TM10, the variations seen in the EPR accessibility studies of the extracellular part of the *so‐called* static TM10 highlight a rather dynamic region of the protein both in terms of polarity and hydrophobicity, which is readily influenced by the position, curvature, and composition of the surrounding membrane. Finally, it should be taken into consideration that crystallisation techniques can only be performed for membrane transporters isolated from their native membranes by solubilisation into detergent micelles. Such non‐native conditions could mask and/or prevent global/local dynamic movements of certain protein regions. Also, since the spin label accessibility data in proteoliposomes are compared against crystallographic LeuT models generated in detergent solubilised micelles, any divergences or discrepancies concerning the structural dynamics of the extracellular part of TM10 during the transport cycle could be potentially attributed to the distinct nature of the lipid bilayer. Previous EPR studies on the sodium‐coupled aspartate transporter, Glt_Ph_, revealed significant heterogeneity of the structural dynamics between detergent micelles and reconstituted proteoliposomes with the dominance of certain conformational states depending on the respective membrane environment (Georgieva et al. 2013; Hanelt et al. 2013).

## 5 Conclusions and Outlook

In this study, an attempt to assign distinct ion/substrate‐dependent conformational states to LeuT using cw‐EPR spectral simulations, which, in turn, can quantify the rotameric spin‐label conformations comprising the cw EPR spectra, was made. Since EPR spectroscopy is a rather sensitive method to study protein dynamics, the information generated from spectral simulations alone may not directly reflect actual ion/substrate‐dependent conformational transitions of LeuT, but can be used as a reliable initial indicator for further experimental designs.

This work describes establishing a robust methodology to study spin label dynamics and accessibility using a combination of EPR spectral simulations and cw EPR power saturation studies. By determining both Π (O_2_) and Π (Ni‐EDDA) accessibility parameters, as well as the polarity index, Φ, especially in proteoliposomes the conformational states in which K398C is accessible to a solvent‐exposed cavity on the EV, or buried within the protein's TM core or is more orientated towards the lipid membrane‐solvent interface have been observed and characterised in detail. Moreover, the spin label accessibility of LeuT K398C complements the cw EPR spectral simulations with a striking example being the thorough analysis of the *OOcL* and *OOcA* states. The cw EPR spectral simulations reveal the dominance of a single mobile component in both ion/substrate‐occluded states observed in both membrane environments, while the respective accessibility studies on these specific configurations provide complementary dynamic, structural information. The combination of these two EPR methods allowed the identification of ‘subtle’ dynamic transitions of the extracellular part of TM10.

The choice of K398C, located near the membrane extracellular interface, permitted EPR, as a very sensitive technique, to be able to monitor where differences in lipid composition between the two environments studied reveal a mixture of conformations in the *IOpApo apo* state. Especially very subtle changes in the label's local environment are mainly but not solely dependent on various ion/substrate‐dependent conformational alterations. Further complexity arises when studying reconstituted membrane transporters in proteoliposomes since not all embedded membrane protein molecules adopt the same orientation, with a significant fraction adopting an ‘inside‐out’ position (Mulligan and Mindell 2017; Jin et al. 2014; Mulligan et al. 2014; Ryan et al. 2009). Overall, similar trends between detergent solubilised micelles and reconstituted proteoliposomes were observed, although the relative magnitudes of these observations proved to be different.

In summary, it has proven rather challenging to extract a holistic accessibility profile for K398C since its location at the membrane interface results in fluctuations and/or movements creating complex ensembles or rotameric populations that can relocate between the surface and the protein/lipid interior in distinct hydrophilic and hydrophobic subregions, or combinations of both. Such considerations suggest extra caution should be taken when interpreting EPR data of cysteine variants to support dynamic models for membrane transport. The recent use of genetically engineered double‐histidine motifs may provide a novel alternative for future EPR‐based investigations since paramagnetic divalent metal ions are an interesting target for coarse‐grained structural investigations of complex systems such as membrane proteins (van Wonderen et al. 2013; Käss et al. 2000).

## Author Contributions

**Petros Tsalagradas:** conceptualization, investigation, writing – original draft, writing – review and editing, formal analysis, data curation, visualization. **Callum Eke:** writing – original draft, investigation, conceptualization, writing – review and editing, formal analysis, data curation, visualization. **Courtney Andrews:** investigation, formal analysis, data curation. **Fraser MacMillan:** conceptualization, funding acquisition, writing – review and editing, project administration, formal analysis, supervision, data curation, methodology, validation.

## Ethics Statement

No animal/human studies are involved in this work.

## Conflicts of Interest

The authors declare no conflicts of interest.

### Peer Review

The peer review history for this article is available at https://www.webofscience.com/api/gateway/wos/peer‐review/10.1111/jnc.70034.

## Supporting information


Data S1.

## Data Availability

The data that support the findings of this study are available from the corresponding author upon reasonable request.
