# Supplementary material for: Exploring the Structural Dynamics of LeuT Using EPR Spectroscopy: A Focus on Transmembrane Helix 10
Source: J Neurochem. 2025 Mar 7;169(3):e70034. doi: 10.1111/jnc.70034 (PMC11886772; doi:10.1111/jnc.70034)
Supplement: Supplementary file 1 — Data S1. [file JNC-169-0-s001.docx]

**Exploring the structural dynamics of LeuT using EPR spectroscopy: A focus on transmembrane helix 10**

Petros Tsalagradas^‡^, Callum Eke^‡^, Courtney Andrews & Fraser MacMillan*

*Henry Wellcome Unit for Biological EPR, University of East Anglia, Norwich, NR4 7TJ, U.K.*

^‡^ Both authors contributed equally to this work.

*Corresponding author

Email fraser.macmillan@uea.ac.uk

Address: School of Chemistry, University of East Anglia, Norwich Research Park, Norwich, NR4 7TJ, UK. Telephone + 44 1603 592766. Fax + 44 1603 59 2003

# **Abbreviations:**

**EPR** Electron Paramagnetic Resonance spectroscopy

**FRET** Förster resonance energy transfer

**IOc** Inward facing Occluded

**IOcF** Inward facing Occluded with phenylalanine (PDB: 6XWM)

**IOp** Inward facing Open

**IOpApo** Inward facing Open Apo (PDB: 3TT3)

**LeuT** Leucine Transporter

**MD** Molecular Dynamics

**OOc** Outward facing Occluded

**OOcA** Outward facing Occluded with alanine (PDB: 3F48)

**OOcL** Outward facing Occluded with leucine (PDB: 2A65)

**OOp** Outward facing Open (PDB: 3TT1)

**OOpW** Outward facing Open with tryptophan (PDB: 3F3A)

**OPM** Orientations of Proteins in Membranes

**OReApo** Outward facing Return Intermediate Apo (PDB: 5JAE)

**PDB** Protein Data Bank

**RRID** Research Resource Identifier (see scicrunch.org)

**SDSL** Site–directed spin labelling

**TM** Transmembrane Helix

**Supplementary data**

**Table of contents**

**page**

1. Transport cycle of LeuT	4
2. Expression, purification, and spin–labelling of LeuT K398C	6
3. Continuous–wave EPR spectroscopy of LeuT in detergent micelles and proteoliposomes	10
4. Computational EPR spin labelling of LeuT K398C based on a s library approach	17
5. Continuous–wave EPR power saturation profiles of LeuT K398C in detergent micelles and proteoliposomes	21
6. References	24

# **1. Transport cycle of LeuT**

**Figure S1. Illustrative description of the LeuT transport cycle using known crystal structures.** **(A)** the **OOp** state is stabilised by the binding of two Na^+^ ions and a substrate is expected to bind to S1 isomerising the transporter to an **OOcL** conformation **(B)**. Further structural alterations lead LeuT to a **IOcF** state **(C)** followed by release of substrate and Na^+^ ions and adopting a **IOpApo** state **(D)**. Finally, LeuT completes the cycle by transitioning back to a more stable outward–open state through a **OReApo** intermediate conformation **(E)** and is ready to repeat the cycle. The reporter molecule used in this study (MTSL), attached at position K398 is indicated in cyan as well the approximate position of the membrane plane (grey lines). The figure was adapted from^3^.

# **2. Expression, purification, and spin–labelling of LeuT K398C**

Recombinant plasmids containing the LeuT K398C construct were used to transform chemically competent C41 DE3 *Escherichia coli* bacterial cells (10 ng μl^–1^, OverExpress™ C41(DE3) Chemically Competent Cells, Sigma Aldrich, cat. no. CMC0017) and prepare glycerol expression stocks. Cells were grown in nutrient broth (NB) medium in the presence of 100 μg mL^–1^ ampicillin (Sigma Aldrich, cat. no. A9518) until an optical density (OD) of 0.3 at 600 nm (37°C, 180 rpm) was reached before incubation temperature was lowered to 18°C. When the OD reached 0.6, LeuT K398C overexpression was induced via addition of isopropyl β–D–1–thiogalactopyranoside (IPTG) (210 μM, Dioxane–Free, Formedium, cat. no. IPTG025) and carried out overnight for 20 – 22 hours (18°C, 180 rpm). Cells were pelleted by centrifugation (6000 rcf, 12 min, Avanti J–26S XP, Beckman Coulter) and re–suspended in lysis buffer (50 mM HEPES pH: 7.5 (Formedium, cat. no. HEPES10), 300 mM KCl (Sigma Aldrich, cat. no. P9541), 30% glycerol (Sigma Aldrich, cat. no. G7757), 5 mM MgCl_2_ (Sigma Aldrich, cat. no. M8266), 1 EDTA–free protease inhibitor cocktail tablet (Roche, cat. no. 04693159001), 1 mM TCEP (Sigma Aldrich, cat. no. C4706), 200 μg mL^–1^ lysozyme (Sigma Aldrich, cat. no. 62971), 10 μg mL^–1^ DNase (Roche, cat. no. 11284932001)) with a 3:1 lysis buffer:cell pellet ratio. Cell lysis was performed using a high–pressure LM20 Microfluidizer™ processor (Microfluidics) for four cycles at 18,000 psi. After lysis, phenylmethylsulfonyl fluoride (PMSF, Sigma Aldrich, cat. no. PMSF–RO) was added to the lysate to a concentration of 1.33 mM. Subsequent removal of cellular debris was conducted via centrifugation (4000 rcf, 20 min, 4°C, Avanti J–25, Beckman Coulter) with the resulting supernatant being further subjected to ultracentrifugation (^~^125.440 rcf, 2 h, 4°C, Beckman L 90 K Optima Ultra Centrifuge). The pellet containing the membrane fragments was re–suspended in solubilisation buffer (50 mM HEPES pH: 7.5, 300 mM KCl, 30% glycerol) and n–dodecyl–β–D–maltoside (DDM, Glycon Biochemicals GmbH, cat. no. D97002) detergent was added to a final concentration of 1.5% following incubation for 2 h at 4°C by gentle agitation to solubilise LeuT K398C in micelles. Afterwards, the solution was subjected to additional ultracentrifugation (~80,282 rcf, 30 min) to separate any insoluble protein aggregates.

The solution containing solubilised LeuT K398C in DDM micelles was added to a pre–equilibrated Ni–IDA Immobilised Metal Affinity Chromatography (IMAC) resin (5 mL, His60 Ni Superflow Resin, Clontech Laboratories, Inc., Takara Bio, cat. no. 635660) with IMAC buffer (20 mM HEPES pH: 7.5, 200 mM KCl, 20% glycerol, 0.05% DDM), imidazole (pH: 8.0, Sigma Aldrich, cat. no. 56750) was added to a final concentration of 40 mM, and the mixture was left to incubate for 2 h at 4°C under gentle agitation to allow for protein binding. A total of 4 wash steps were performed using a total of 32 column volumes (CV) of wash buffer A (IMAC buffer supplemented with 40 mM of imidazole). Spin–labelling of LeuT K398C was performed on the IMAC column after the final wash step by adding 1–Oxyl–2,2,5,5–tetramethyl–Delta–3–pyrroline–3–methyl Methanethiosulfonate (MTSL, Toronto Research Chemicals Inc., cat. no. TRC–O875000) to a final concentration of 1 mM and the mixture was incubated overnight (16 – 18 h) at 4°C under gentle agitation in the absence of light. The following day, the spin–labelled resin solution was transferred to a gravity–flow column and a final wash was performed using 11 CV of wash buffer B (IMAC buffer supplemented with 90 mM of imidazole). The protein was eluted in 9 1 mL fractions using elution buffer (IMAC buffer supplemented with 300 mM of imidazole) and its purity determined with SDS–PAGE gel electrophoresis. Fractions containing the largest quantities of eluted LeuT were pooled together and concentrated using an Amicon Ultra–4 Centrifugal Unit (100 kDa MWCO, Millipore, cat. no. UFC8100) to a final volume of 2.5 mL before buffer exchange (PD10 Desalting Columns, Sephadex™ G–25 resin, Cytiva, cat. no. GE17–0851–01) with IMAC buffer to remove any remaining imidazole. Protein concentration was estimated by measuring the optical absorbance at 280 nm (A_280_) using a NanoDrop 1000 Spectrophotometer (ThermoFisher Scientific) using an extinction coefficient at 280 nm (113,340 cm ^–1^M ^–1^)^4^. Purified LeuT K398C was either flash frozen and stored at –80°C or further concentrated using an Amicon Ultra–4 Centrifugal Unit (100 kDa MWCO) to a final concentration (80 – 120 μΜ) for the EPR experiments. Identical protein overexpression and purification protocols were performed for the LeuT cysteine mutant, R86C.

**SDS–PAGE electrophoresis**

Protein samples (15 μL) were collected at various points during purification and after sample concentration before the addition of 4x–concentrated loading dye (5 μL, 1M Tris–HCl pH: 6.8 (Formedium, cat. no. TRIS01), 8 % (w/v) SDS (Sigma Aldrich, cat. no. L3771), 10% glycerol, 572 mM β–mercaptoethanol (β–ME, Sigma Aldrich, cat. no. M3148), 0.5M EDTA (Sigma Aldrich, cat. no. E5134), 0.08 % (w/v) bromophenol blue (Sigma Aldrich, cat. no. B–5525)). Finally, 4 μl of protein marker (EZ–Run™ Pre–Stained *Rec* Protein Ladder, Fisher Bioreagents™, cat. No. BP3603–500) and 15 μL of each sample were loaded onto a 12 % SDS–PAGE gel and run for 1 h at a constant 190 V followed by gel staining with Coomassie (ReadyBlue^™^ Protein Gel Stain, Sigma Aldrich, cat. no. RSB) for 30 min at ambient temperature.

**Purification and efficient spin labelling of LeuT K398C with MTSL**

Protein purity was determined using SDS–PAGE electrophoresis (**Figure S3**). After washing (W1–W5) to remove any unbound membrane proteins (impurities) spin–labelled LeuT K398C was eluted (E0–E8) revealing a distinct band at ~43–45 kDa. LeuT’s predicted, theoretical molecular size is ~58 kDa^5^ and the difference observed here is attributed to a “gel–shifting” effect, common among membrane proteins with larger hydrophobic domains, which can bind more SDS molecules and hence run faster on a gel^6^. Final protein concentrations of spin–labelled LeuT K398C were ~0.47 mg ml^–1^ (8 μM) with a final protein yield of 0.47 mg L^–1^ of bacterial culture.

|  |  |
| --- | --- |
| **Figure S2.** **(A)** SDS–PAGE analysis of LeuT K398C purification steps after binding on the Ni–IDA IMAC resin. Fractions W1 – W4: washes of the LeuT–bound Ni^2+^ resin using imidazole diluted in wash buffer (40 mM); W5, a final wash step with 90 mM imidazole. Fractions E0 – E8 show protein elution with 300 mM imidazole and “M” indicates protein markers. **(B)** LeuT K398C concentration using an Amicon–4 Ultra centrifugal unit (100 MWCO). “FT” show the flow–through fraction of the ultrafiltration process while “LeuT K398C +SL” represents concentrated, spin–labelled LeuT K398C. | |

Recombinant LeuT cysteine mutant, K398C, was purified via a novel single IMAC/SDSL step during the purification procedure resulting in higher spin–labelling efficiency. It has been shown previously that a cysteine mutation at position 398 does not impair LeuT function and ligand binding capacity^7^. Similar functional assays on LeuT variants with attached spin labels have also shown no impairment (data not shown). The efficiency of spin labelling LeuT K398C was determined to be ~60%.

The single cysteine variant, K398C, was successfully overexpressed, isolated, purified and efficiently spin–labelled with the MTSL nitroxide label using a single IMAC purification step and Ni^2+^ chromatography column. Overall purity was very high (**Figure S2**) and only miniscule amounts of LeuT K398C were detected in the flow–through during the concentration process using a 100 kDa molecular weight cut–off filter, which is expected since the theoretical, total molecular weight of LeuT (58 kDa) embedded in a detergent–solubilised micelle (~70kDa) is ~128 kDa. The results of LeuT K398C purification and spin–labelling efficiency (^~^60%) correlate well with a previous study on LeuT K398C using fluorescent probes for transition metal ion fluorescence resonance energy transfer (FRET) experiments^7^. In addition, functional assays clearly demonstrate that the cysteine mutation at position 398 doesn’t impair protein function and binding capacity of leucine compared to wild–type LeuT^7, 8, 9^. In addition, functional assays performed on LeuT cysteine variants (including R86C) with and without an attached MTSL spin label do not reveal any structural and/or functional perturbations (unpublished data not shown) compared to wild–type LeuT. Therefore, since the average size of a nitroxide label is much smaller than that a bulky fluorescent probe and only resembles the size of a naturally occurring aromatic amino acid, it can be inferred that its attachment at position K398C does not impair the protein’s structure and function. Finally, extensive transport and functional assays have been conducted on liposome–reconstituted LeuT K398C labelled with a fluorescent probe and showed that the transporter was fully functional^8, 9^. Similarly, since fluorescent probes are bulkier in size compared to nitroxide spin–labels (size of a naturally occurring aromatic amino acid as mentioned above), we can infer that there are no structural perturbations caused by the attached nitroxide spin–label on LeuT K398C in both detergent–solubilised micelles and reconstituted proteoliposomes.

# **3. Continuous–wave EPR spectroscopy of LeuT in detergent micelles and proteoliposomes**

The X–band cw EPR spectra of a nitroxide label bound on LeuT at K398C and R86C (**Figure S3A**) and R86C under various ion/ligand–dependent conformational states were recorded at ambient temperature in detergent–solubilised micelles (**Figure S3B**) which clearly indicate that position R86C is static in the various states of the transport cycle, clearly different from K398C. Similar effects are also seen in the power saturation data (see section 5).

**Figure S3. (A)** X–band cw EPR spectra of MTSL bound on LeuT K398C and R86C under the **IOpApo** conformation in detergent–solubilised (DDM) micelles recorded at ambient temperature. A small contribution of unspecific free label (<~2–3%) remains in all protein–bound sample spectra (*) while the unattached MTSL in buffer is presented as the lowest trace. **(B)** X–band cw EPR spectra of MTSL bound on LeuT R86C under all five conformational configurations in detergent–solubilised (DDM) micelles recorded at ambient temperature. All plots were generated in MATLAB^®^. Experimental conditions were as described in the main paper (see materials and methods).

***IOpApo****: Inward facing Open Apo,* ***OOp****: Outward facing Open (Na^+^–bound),* ***OOcL****: Outward facing Occluded with leucine (Na^+^/leucine–bound),* ***OOcA****: Outward facing Occluded with alanine (Na^+^/alanine–bound),* ***OOpW****: Outward facing Open with tryptophan (Na^+^/tryptophan–bound)*

The X–band cw EPR spectra of a nitroxide label bound on LeuT K398C under various ion/ligand–dependent conformational states were recorded at cryogenic temperatures (10 and 50 K) in detergent–solubilised micelles (**Figure S4**).

**Figure S4.** **(A)** X–band cw EPR spectra of MTSL bound on LeuT K398C in detergent–solubilised micelles recorded at cryogenic temperature (50 K) under: the **IOpApo**, **OOp**, **OOcL**, **OOcA**, and **OOpW** conformations. **(B)** X–band cw EPR spectrum of LeuT K398C poised under the **OOp** conformation which was recorded at two distinct cryogenic temperatures (10 and 50 K). Importantly, both measurements were performed on the same LeuT K398C sample for consistency. All EPR spectral simulations are shown in dotted lines, were performed using the MATLAB^®^–based EasySpin software, and all plots were generated in MATLAB^®^. Experimental conditions were as described in the main paper (see materials and methods).

***IOpApo****: Inward facing Open Apo,* ***OOp****: Outward facing Open (Na^+^–bound),* ***OOcL****: Outward facing Occluded with leucine (Na^+^/leucine–bound),* ***OOcA****: Outward facing Occluded with alanine (Na^+^/alanine–bound),* ***OOpW****: Outward facing Open with tryptophan (Na^+^/tryptophan–bound).*

For low–temperature EPR studies (**Figure S4**), K398C spin labelled LeuT (70μM) was added to 4 mm suprasil quartz tubes (Wilmad SQ–707; Wilmad–LabGlass, Vinland, NJ, USA), poised in the respective state (***IOpApo***, ***OOp***, ***OOcA***, ***OOcL***, ***OOpW***) and flash frozen in liquid nitrogen. X–band cw–EPR spectra were recorded on a Bruker eleXsys E560 spectrometer using a cylindrical Bruker cavity (ER4122SHQE) equipped with an Oxford helium cryostat (ESR900). Experimental parameters: microwave power, 10 mW; field modulation amplitude, 0.1 mT; field modulation frequency, 100 kHz; temperatures, 10 and 50K. The measured spectra were corrected for an offset against a known g standard [DPPH, 1,1–diphenyl–2–picrylhydrazyl, g = 2.00360 ± 0.00002]. Spectral simulations were performed using the Matlab^®^–based EasySpin package.

| **Table S1.** EPR parameters determined from the low–temperature spectral simulations LeuT K398C in detergent micelles. | | |
| --- | --- | --- |
| State | g_ZZ_* | A_ZZ_ / mT |
| ***IOpApo*** | 2.00184 | 3.575 |
| ***OOp*** | 2.00193 | 3.585 |
| ***OOcL*** | 2.00189 | 3.635 |
| ***OOcA*** | 2.00189 | 3.650 |
| ***OOpW*** | 2.00200 | 3.685 |

* values corrected against DPPH

The EPR spectral simulations at ambient temperature requires a large set of variables (see **Table S2**), however by using low temperature (50K) EPR key essential parameters can be determined independently and then held fixed in subsequent fitting routines. By using an EPR standard (DPPH) the g_ZZ_ parameter can be accurately determined while the A_ZZ_ parameter can simply be read off from the low temperature spectrum (or from spectral simulations using the Easyspin ‘pepper’ function in Matlab^®^), see **Table S1**. Together with typical g- (DG(g_xx_-g_yy_) and DG(g_yy_-g_zz_) and hyperfine-tensor anisotropies^10^ determination of accurate rotational correlation times (*t_c_*) is possible.

| **A** | **Species** | **g_xx_*** | **g_yy_*** | **g_zz_*** | **g_iso_*** | **A_xx_ / mT** | **A_yy_ / mT** | **A_zz_ / mT** | **A_iso_ / mT** | **Lwpp / mT** | **t_c_ /ns** | **Weight (%)** |
| --- | --- | --- | --- | --- | --- | --- | --- | --- | --- | --- | --- | --- |
| ***IOpApo*** | 1 | 2.00825 | 2.00656 | 2.00232 | 2.00571 | 0.64 | 0.51 | 3.54 | 1.56 | 0.21 | 50.2 | 34.1 |
|  | 2 | 2.00856 | 2.00622 | 2.00245 | 2.00574 | 0.46 | 0.75 | 3.55 | 1.59 | 0.18 | 6.9 | 63.9 |
| ***OOp*** | 1 | 2.00810 | 2.00658 | 2.00251 | 2.00573 | 0.65 | 0.51 | 3.55 | 1.57 | 0.19 | 48.8 | 24.5 |
|  | 2 | 2.00833 | 2.00683 | 2.00252 | 2.00589 | 0.45 | 0.75 | 3.53 | 1.58 | 0.14 | 7.1 | 70.2 |
| ***OOcL*** | 1 | 2.00899 | 2.00640 | 2.00252 | 2.00597 | 0.58 | 0.60 | 3.63 | 1.60 | 0.20 | 7.6 | 93.7 |
| ***OOcA*** | 1 | 2.00909 | 2.00644 | 2.00250 | 2.00601 | 0.58 | 0.59 | 3.65 | 1.61 | 0.10 | 6.8 | 95.0 |
| ***OOpW*** | 1 | 2.00791 | 2.00624 | 2.00234 | 2.00550 | 0.66 | 0.52 | 3.53 | 1.57 | 0.20 | 47.5 | 16.3 |
|  | 2 | 2.00850 | 2.00585 | 2.00250 | 2.00562 | 0.46 | 0.75 | 3.51 | 1.57 | 0.21 | 11.1 | 80.7 |

| **B** | **Species** | **g_xx_*** | **g_yy_*** | **g_zz_*** | **g_iso_*** | **A_xx_ / mT** | **A_yy_ / mT** | **A_zz_ / mT** | **A_iso_ / mT** | **Lwpp / mT** | **t_c_ /ns** | **Weight (%)** |
| --- | --- | --- | --- | --- | --- | --- | --- | --- | --- | --- | --- | --- |
| ***IOpApo*** | 1 | 2.00863 | 2.00656 | 2.00236 | 2.00585 | 0.64 | 0.52 | 3.45 | 1.54 | 0.25 | 56.0 | 34.2 |
|  | 2 | 2.00851 | 2.00624 | 2.00247 | 2.00574 | 0.45 | 0.74 | 3.49 | 1.56 | 0.20 | 6.5 | 60.1 |
| ***OOp*** | 1 | 2.00900 | 2.00680 | 2.00249 | 2.00610 | 0.62 | 0.50 | 3.45 | 1.53 | 0.21 | 106.3 | 16.3 |
|  | 2 | 2.00860 | 2.00645 | 2.00245 | 2.00583 | 0.50 | 0.74 | 3.65 | 1.63 | 0.20 | 4.1 | 81.5 |
| ***OOcL*** | 1 | 2.00859 | 2.00595 | 2.00245 | 2.00566 | 0.61 | 0.58 | 3.62 | 1.61 | 0.26 | 6.0 | 95.8 |
| ***OOcA*** | 1 | 2.00891 | 2.00670 | 2.00248 | 2.00603 | 0.64 | 0.55 | 3.64 | 1.61 | 0.19 | 5.1 | 94.4 |
| ***OOpW*** | 1 | 2.00932 | 2.00682 | 2.00248 | 2.00621 | 0.61 | 0.52 | 3.58 | 1.57 | 0.16 | 66.9 | 15.9 |
|  | 2 | 2.00875 | 2.00700 | 2.00245 | 2.00607 | 0.50 | 0.66 | 3.59 | 1.58 | 0.20 | 4.9 | 82.0 |

**Table S2:** EPR parameters determined from the ambient temperature spectral simulations of LeuT K398C in (A) detergent micelles and (B) reconstituted into proteoliposomes.

* values corrected against DPPH

Similarly, the X–band cw EPR spectra of LeuT R86C under the same conformational states as with K398C were recorded at identical cryogenic temperatures (10 and 50 K) in detergent–solubilised micelles for consistency (**Figure S5**).

**Figure S5.** **(A)** X–band cw EPR spectra of MTSL bound on LeuT R86C in detergent–solubilised micelles recorded at cryogenic temperature (50 K) under: the ***IOpApo***, ***OOp***, ***OOcL***, ***OOcA***, and ***OOpW*** conformations. **(B)** X–band cw EPR spectrum of LeuT R86C poised under the ***OOcA*** conformation which was recorded at two distinct cryogenic temperatures (10 and 50 K). Importantly, both measurements were performed on the same LeuT R86C sample for consistency. All EPR spectral simulations are shown in dotted lines, were performed using the MATLAB^®^–based EasySpin software, and all plots were generated in MATLAB^®^. Experimental conditions were as described in the main paper (see materials and methods).

***IOpApo****: Inward facing Open Apo,* ***OOp****: Outward facing Open (Na^+^–bound),* ***OOcL****: Outward facing Occluded with leucine (Na^+^/leucine–bound),* ***OOcA****: Outward facing Occluded with alanine (Na^+^/alanine–bound),* ***OOpW****: Outward facing Open with tryptophan (Na^+^/tryptophan–bound).*

**Figure S6** displays the weighted individual spectral components that make up the overall spectral simulations of cw–EPR datasets for the following conformational states of LeuT K398C in both detergent–solubilised micelles and reconstituted proteoliposomes: ***OOp***, ***OOcL***, ***OOcA***, ***OOpW***.

| **Detergent micelles** | **Proteoliposomes** |
| --- | --- |
|  |  |
|  |  |
|  |  |
|  |  |
| **Figure S6**. Representation of all individual cw spectral components for LeuT K398C conformations of: **OOp (A, B)**, **OOcL (C, D)**, **OOcA (E, F)** and **OOpW (G, H)** in detergent–solubilised (DDM) micelles (left; A, C, E, G) and reconstituted proteoliposomes (right; B, D, F, H) with the combined simulation output comprising of the same spectra as presented in **Figure 2A–B** (dotted lines), respectively, and generated by the MATLAB^®^–based EasySpin toolbox. The intensities of the individual spectra of all bound species are given in **Table 1**, while the contribution of the unbound species is ^~^2–6%.  ***OOp****: Outward facing Open (Na^+^–bound),* ***OOcL****: Outward facing Occluded with leucine (Na^+^/leucine–bound),* ***OOcA****: Outward facing Occluded with alanine (Na^+^/alanine–bound),* ***OOpW****: Outward facing Open with tryptophan (Na^+^/tryptophan–bound)* | |

# **4. Computational EPR spin labelling of LeuT K398C based on a rotamer library approach**

The three–dimensional space occupied by each rotamer ensemble attached at position 398 in three key conformations of LeuT are depicted in **Figure S8**. Although a total of five conformational states of LeuT K398C were studied here, only three are presented in **Figure S7** for clarity. The MTSL rotameric populations on LeuT K398C structural models under the various, distinct conformational configurations studied here are presented in **Figure S8** while **Table S3** describes the number of MTSL rotamer populations for all the known conformations.

**Figure S7. Visualisation of in silico EPR analyses.** The spin label rotamers of MTSL attached to K398C together with the gating helices, TM10 and TM1, are highlighted while the rest of the LeuT backbone is set to transparent for clarity. More specifically, the **OOp** (3TT1), **OOcL** (2A65), and **IOpApo** (3TT3) states are superimposed and depicted in green, cyan, and red, respectively. The MTSL rotamers were computationally attached using MMM and all structural models of LeuT K398C were visualised with ‘UCSF Chimera’.

***IOpApo****: Inward facing Open Apo,* ***OOp****: Outward facing Open (Na^+^–bound),* ***OOcL****: Outward facing Occluded with leucine (Na^+^/leucine–bound)*

|  |  |
| --- | --- |
|  |  |
|  |  |
|  |  |
|  |  |
| **Figure S8. Visualisation of the *in silico* EPR analyses of LeuT K398C**. The spin label rotamers of MTSL attached to K398C under the five studied conformational states are shown from two different angles, a perpendicular zoom to the membrane plane (A, C, E, G, I) and a panoramic view of the rotameric ensembles with the Na^+^ ions and the corresponding substrates (L–leucine, L–alanine) and inhibitor (L–tryptophan) highlighted, where applicable (B, D, F, H, J). The rest of the protein backbone was set to transparent for all LeuT crystal structures while residues 471–484 were omitted from the structural models shown in A, C, E, G, I for clarity. More specifically, the ***IOpApo*** (3TT3, red), ***OOp*** (3TT1, green), ***OOcL*** (2A65, cyan), ***OOcA*** (3F48, orange) and ***OOpW*** (3F3A, purple) states were superimposed, and the different visual angles of the attached MTSL rotamers are presented. Sodium ions are depicted as blue spheres while the two substrate molecules, L–leucine and L–alanine, as well as the inhibitor, L–tryptophan, are shown as spheres in grey, yellow, and magenta, respectively. The MTSL rotamers were computationally attached using MMM and all structural models were visualised and rendered with ‘UCSF’ Chimera.  ***IOpApo****: Inward facing Open Apo,* ***OOp****: Outward facing Open (Na^+^–bound),* ***OOcL****: Outward facing Occluded with leucine (Na^+^/leucine–bound),* ***OOcA****: Outward facing Occluded with alanine (Na^+^/alanine–bound),* ***OOpW****: Outward facing Open with tryptophan (Na^+^/tryptophan–bound).* | |

| **Table S3.** *In silico* EPR rotamer library analysis of a computationally attached nitroxide spin probe (MTSL) on LeuT crystal structure models using MMM. | | | | |
| --- | --- | --- | --- | --- |
| **LeuT Conformation** | **Ion/ligand** | **PDB entry** | **K398C Rotamers** | **R86C Rotamers** |
| Inward – Open (***IOpApo***) | Apo | 3TT3 ^10^ | 39 | 133 |
| Outward – Return (***OReApo***) | Apo | 5JAE ^11^ | 53 | 137 |
| Outward – Open (***OOp***) | 2Na^+^ | 3TT1 ^10^ | 57 | 140 |
| Outward – Occluded (***OOcL***) | 2Na^+^ / Leu | 2A65 ^12^ | 65 | 134 |
| Outward – Occluded (***OOcA***) | 2Na^+^ / Ala | 3F48 ^13^ | 60 | 138 |
| Inward – Occluded (***IOcF***) | 2Na^+^ / Phe | 6XWM ^14^ | 31 | 133 |
| Outward – Open (***OOpW***) | 2Na^+^ / Trp | 3F3A ^13^ | 56 | 131 |

# **5. Continuous–wave EPR power saturation profiles of LeuT K398C in detergent micelles and proteoliposomes**

All EPR microwave power saturation experiments and data analysis were performed as described previously^15, 16, 17, 18^.

The cw EPR power saturation profiles of spin labelled LeuT K398C in the ***OOp***, ***OOcA***/***OOcL*** and ***OOpW*** conformational states reveal clear differences in the presence of both paramagnetic quenchers (O_2_, Ni–EDDA) as compared to diamagnetic N_2_(g) in both detergent micelles and proteoliposomes (**Figure S9**).

All cw EPR power saturation curves of LeuT K398C in both detergent–solubilised micelles and reconstituted proteoliposomes were fitted using the peak–to–peak intensity of the central resonance line plotted against $\log\left( \sqrt{P} \right)$ and equation 1 to determine a corresponding $P_{\mathbf{½}}$ value using MATLAB^®^:

$Y^{\boldsymbol{'}}= \frac{I\sqrt{P}}{\left[ 1+\left( 2^{\frac{1}{\varepsilon}}-1 \right)P/P_{\mathbf{½}} \right]^{\varepsilon}}$	(1)

Where the inhomogeneity parameter $\varepsilon$ takes the value 1.5 in the homogeneous limit and 0.5 in the inhomogeneous limit. Usually, $\varepsilon$ is not known beforehand and is treated as a fit parameter. The other fit parameters are *I*, which is the slope of the amplitude increase with the square root of microwave power in the linear regime, and *P*_½_, which is the half–saturation power. More precisely, *P*_½_ is the incident mw power where *Y* ’ is reduced to half of its unsaturated value.

The accessibility parameter, $\Pi$, for each state was calculated using equation 2 using a reference powder sample, 2,2–diphenyl–1–picrylhydrazyl (DPPH), recorded using identical EPR experimental parameters, with $P_{\mathbf{½}}$ and the respective average linewidth, *ΔH_pp_*.

$\Pi\left( X \right)= \frac{{\Delta P}_{½}\left( x \right)/{\Delta H}_{pp}(x)}{P_{½}(DPPH)/{\Delta H}_{pp}(DPPH)}$	(2)

where ${\Delta P}_{½}\left( x \right)$ *=* $P_{½}\left( x \right)$*–* $P_{½}$*(N_2_)* and *ΔH_pp_(*$x$*) (*$x$ is either O_2_ or Ni–EDDA*)*. The polarity index, $\Phi$, was estimated for each conformation of LeuT K398C using equation 3:

$\Phi= \ln\left[ \frac{{\Pi(O}_{2})}{\Pi(NiEDDA)} \right]$	(3)

**Figure S9.** Normalised cw EPR power saturation curves of LeuT K398C in detergent–solubilised micelles (left) and proteoliposomes (right) under **OOp** **(A)**, **OOcL** **(B)**, **OOcA** **(C)**, and **OOpW** conformations **(D)**. The cw EPR spectra used to generate the saturation profiles were recorded in the presence of O_2_ (blue, circles), N_2_ (black, squares), and 50 mM Ni–EDDA (red, triangles). All signal intensities (arbitrary units) were plotted against the square root of the incident microwave power and all plots were generated in MATLAB^®^.

***OOp****: Outward facing Open (Na^+^–bound),* ***OOcL****: Outward facing Occluded with leucine (Na^+^/leucine–bound),* ***OOcA****: Outward facing Occluded with alanine (Na^+^/alanine–bound),* ***OOpW****: Outward facing Open with tryptophan (Na^+^/tryptophan–bound).*

Finally, the cw EPR power saturation curves for LeuT R86C in detergent–solubilised micelles were generated by following similar EPR data acquisition and data analysis to LeuT K398C and the results are presented in **Table S4** and **Figure S10**.

| **Table S4**. EPR accessibility profiles of spin labelled LeuT R86C in detergent-solubilised micelles. | | | | |
| --- | --- | --- | --- | --- |
| **Detergent micelles (DM)** | | | | |
| State | P_½_ (mW)  (O_2_) | P_½_ (mW)  (Ni-EDDA) | Accessibility  $\Pi$ (O_2_) | Accessibility  $\Pi$ (Ni-EDDA) |
| ***IOpApo*** | 12.64 | 12.96 | 0.691 | 0.441 |
| ***OOp*** | 13.64 | 11.94 | 0.650 | 0.045 |
| ***OOcL*** | 12.98 | 14.19 | 0.034 | 0.272 |
| ***OOcA*** | 11.93 | 15.76 | 0.166 | 0.942 |

**Figure S10**. Normalised cw EPR power saturation curves of LeuT K398C (left) and R86C (right) in detergent-solubilised micelles under **IOpApo** **(A)**, **OOp** **(B)**, **OOcL** **(C)**, and **OOcA** conformations **(D)**. The cw EPR spectra used to generate the saturation profiles were recorded in the presence of O2 (blue, circles), N2 (black, squares), and 50 mM Ni-EDDA (red, triangles). All signal intensities (arbitrary units) were plotted against the square root of the incident microwave power and all plots were generated in MATLAB^®^.

***IOpApo:*** *Inward facing Open Apo,* ***OOp****: Outward facing Open (Na^+^–bound),* ***OOcL****: Outward facing Occluded with leucine (Na^+^/leucine–bound),* ***OOcA****: Outward facing Occluded with alanine (Na^+^/alanine–bound)*

# **6. References**

1.	Lomize MA, Pogozheva ID, Joo H, Mosberg HI, Lomize AL. OPM database and PPM web server: resources for positioning of proteins in membranes. Nucleic Acids Research. 2012;40(D1):D370-D6.

2.	Pettersen EF, Goddard TD, Huang CC, Meng EC, Couch GS, Croll TI, et al. UCSF ChimeraX: Structure visualization for researchers, educators, and developers. Protein Sci. 2021;30(1):70-82.

3.	Fan J, Xiao Y, Quick M, Yang Y, Sun Z, Javitch JA, et al. Crystal structures of LeuT reveal conformational dynamics in the outward-facing states. J Biol Chem. 2021;296:100609.

4.	Piscitelli CL, Krishnamurthy H, Gouaux E. Neurotransmitter/sodium symporter orthologue LeuT has a single high-affinity substrate site. Nature. 2010;468(7327):1129-32.

5.	Androutsellis-Theotokis A, Goldberg NR, Ueda K, Beppu T, Beckman ML, Das S, et al. Characterization of a functional bacterial homologue of sodium-dependent neurotransmitter transporters. J Biol Chem. 2003;278(15):12703-9.

6.	Rath A, Glibowicka M, Nadeau VG, Chen G, Deber CM. Detergent binding explains anomalous SDS-PAGE migration of membrane proteins. Proceedings of the National Academy of Sciences of the United States of America. 2009;106(6):1760-5.

7.	Sørensen A, Alma L. Optimization of experimental procedure for assessing transition metal ion FRET in LeuT. UCPH Nano-Science. 2018.

8.	Billesbolle CB, Mortensen JS, Sohail A, Schmidt SG, Shi L, Sitte HH, et al. Transition metal ion FRET uncovers K(+) regulation of a neurotransmitter/sodium symporter. Nat Commun. 2016;7:12755.

9.	Schmidt SG, Nygaard A, Mindell JA, Loland CJ. Exploring the K(+) binding site and its coupling to transport in the neurotransmitter:sodium symporter LeuT. Elife. 2024;12.

10.	Krishnamurthy H, Gouaux E. X-ray structures of LeuT in substrate-free outward-open and apo inward-open states. Nature. 2012;481(7382):469-74.

11.	Malinauskaite L, Said S, Sahin C, Grouleff J, Shahsavar A, Bjerregaard H, et al. A conserved leucine occupies the empty substrate site of LeuT in the Na(+)-free return state. Nat Commun. 2016;7:11673.

12.	Yamashita A, Singh SK, Kawate T, Jin Y, Gouaux E. Crystal structure of a bacterial homologue of Na+/Cl--dependent neurotransmitter transporters. Nature. 2005;437(7056):215-23.

13.	Singh SK, Piscitelli CL, Yamashita A, Gouaux E. A competitive inhibitor traps LeuT in an open-to-out conformation. Science. 2008;322(5908):1655-61.

14.	Gotfryd K, Boesen T, Mortensen JS, Khelashvili G, Quick M, Terry DS, et al. X-ray structure of LeuT in an inward-facing occluded conformation reveals mechanism of substrate release. Nat Commun. 2020;11(1):1005.

15.	Klug CS, Feix JB. Methods and applications of site-directed spin labeling EPR spectroscopy. Methods Cell Biol. 2008;84:617-58.

16.	Altenbach C, Greenhalgh DA, Khorana HG, Hubbell WL. A collision gradient method to determine the immersion depth of nitroxides in lipid bilayers: Application to spin-labeled mutants of bacteriorhodopsin. Proceedings of the National Academy of Sciences of the United States of America. 1994;91(5):1667-71.

17.	Altenbach C, Froncisz W, Hyde JS, Hubbell WL. Conformation of spin-labeled melittin at membrane surfaces investigated by pulse saturation recovery and continuous wave power saturation electron paramagnetic resonance. Biophysical Journal. 1989;56(6):1183-91.

18.	Altenbach C, Marti T, Khorana HG, Hubbell WL. Transmembrane protein structure: Spin labeling of bacteriorhodopsin mutants. Science. 1990;248(4959):1088-92.
